# A systems pharmacology-based approach to identify novel Kv1.3 channel-dependent mechanisms in microglial activation

**DOI:** 10.1186/s12974-017-0906-6

**Published:** 2017-06-26

**Authors:** Srikant Rangaraju, Syed Ali Raza, Andrea Pennati, Qiudong Deng, Eric B. Dammer, Duc Duong, Michael W. Pennington, Malu G. Tansey, James J. Lah, Ranjita Betarbet, Nicholas T. Seyfried, Allan I. Levey

**Affiliations:** 1 Department of Neurology, Emory University, 615 Michael Street, Suite 525, Atlanta, GA 30322 USA; 2 Department of Medicine, University of Wisconsin School of Medicine and Public Health, Madison, WI 53726 USA; 3 Department of Biochemistry, Emory University, 615 Michael Street, Suite 525, Atlanta, GA 30322 USA; 4 Peptides International, Louisville, KY 40299 USA; 5 Department of Physiology, Emory University, 615 Michael Street, Suite 525, Atlanta, GA 30322 USA

**Keywords:** Microglia, Neuroinflammation, Potassium channels, Kv1.3, Proteomics

## Abstract

**Background:**

Kv1.3 potassium channels regulate microglial functions and are overexpressed in neuroinflammatory diseases. Kv1.3 blockade may selectively inhibit pro-inflammatory microglia in neurological diseases but the molecular and cellular mechanisms regulated by Kv1.3 channels are poorly defined.

**Methods:**

We performed immunoblotting and flow cytometry to confirm Kv1.3 channel upregulation in lipopolysaccharide (LPS)-activated BV2 microglia and in brain mononuclear phagocytes freshly isolated from LPS-treated mice. Quantitative proteomics was performed on BV2 microglia treated with control, LPS, ShK-223 (highly selective Kv1.3 blocker), and LPS+ShK-223. Gene ontology (GO) analyses of Kv1.3-dependent LPS-regulated proteins were performed, and the most representative proteins and GO terms were validated. Effects of Kv1.3-blockade on LPS-activated BV2 microglia were studied in migration, focal adhesion formation, reactive oxygen species production, and phagocytosis assays. In vivo validation of protein changes and predicted molecular pathways were performed in a model of systemic LPS-induced neuroinflammation, employing antigen presentation and T cell proliferation assays. Informed by pathway analyses of proteomic data, additional mechanistic experiments were performed to identify early Kv1.3-dependent signaling and transcriptional events.

**Results:**

LPS-upregulated cell surface Kv1.3 channels in BV2 microglia and in microglia and CNS-infiltrating macrophages isolated from LPS-treated mice. Of 144 proteins differentially regulated by LPS (of 3141 proteins), 21 proteins showed rectification by ShK-223. Enriched cellular processes included MHCI-mediated antigen presentation (TAP1, EHD1), cell motility, and focal adhesion formation. In vitro, ShK-223 decreased LPS-induced focal adhesion formation, reversed LPS-induced inhibition of migration, and inhibited LPS-induced upregulation of EHD1, a protein involved in MHCI trafficking. In vivo, intra-peritoneal ShK-223 inhibited LPS-induced MHCI expression by CD11b^+^CD45^low^ microglia without affecting MHCI expression or trafficking of CD11b^+^CD45^high^ macrophages. ShK-223 inhibited LPS-induced MHCI-restricted antigen presentation to ovalbumin-specific CD8^+^ T cells both in vitro and in vivo. Kv1.3 co-localized with the LPS receptor complex and regulated LPS-induced early serine (S727) STAT1 phosphorylation.

**Conclusions:**

We have unraveled novel molecular and functional roles for Kv1.3 channels in pro-inflammatory microglial activation, including a Kv1.3 channel-regulated pathway that facilitates MHCI expression and MHCI-dependent antigen presentation by microglia to CD8^+^ T cells. We also provide evidence for neuro-immunomodulation by systemically administered ShK peptides. Our results further strengthen the therapeutic candidacy of microglial Kv1.3 channels in neurologic diseases.

**Electronic supplementary material:**

The online version of this article (doi:10.1186/s12974-017-0906-6) contains supplementary material, which is available to authorized users.

## Background

Neuroinflammation is primarily mediated by microglia, the innate immune cells of the brain. Microglia have opposing pro- and anti-inflammatory roles that when dysregulated, contribute to neuroinflammatory and neurodegenerative disorders [1, 2]. Selective modulators of pro-inflammatory microglia that do not affect homeostatic and neuroprotective microglial functions may therefore be therapeutically relevant. Kv1.3 is a voltage-gated outward-rectifying potassium channel that is expressed by microglia, macrophages, and other immune cells, wherein it regulates membrane potential, calcium signaling, cytokine production, and proliferative responses, and is a promising therapeutic target for autoimmune, neuroinflammatory, and neurodegenerative diseases [3–6]. Selective upregulation of Kv1.3 channels by microglia in disease contexts has been observed in human disease and animal models [4, 7, 8]. Pro-inflammatory microglial functions that are mediated by Kv1.3 channels include direct neurotoxicity, cytokine production, and reactive oxygen species while homeostatic functions of non-activated microglia appear to involve alternative cationic channels [9, 10]. Recent studies have shown that intracellular potassium concentration and potassium efflux mechanisms regulate an immune checkpoint in T cells, raising the possibility that similar roles for Kv1.3 potassium channels in microglia may also exist [11]. However, the molecular and signaling mechanisms regulated by Kv1.3 channels in microglia, particularly in pro-inflammatory-activated states, remain poorly defined. In this study of pro-inflammatory microglial activation, we first determined whether Kv1.3 channels are upregulated by pro-inflammatory stimuli in microglia in a model of acute neuroinflammation induced by lipopolysaccharide (LPS). We then used a systems pharmacology approach to identify Kv1.3 channel-regulated proteins in pro-inflammatory-activated and resting microglial states. These data informed in vitro and in vivo validation studies to unravel novel molecular mechanisms, signaling pathways, and cellular functions that are regulated by Kv1.3 channels.

## Methods

### Reagents

ShK-223, an analog of sea anemone peptide ShK, was used as a highly selective Kv1.3 channel blocker (IC_50_ for Kv1.3 25 ± 14 pM, >10,000-fold more selective for Kv1.3 than neuronal Kv1.1 and Kv1.2 channels) [12]. The dose of ShK-223 for intravenous or intraperitoneal injections was 100 μg/kg. Concentration used for in vitro experiments was 100 nM. ShK-F6CA, a fluorescein-conjugated ShK analog, was purchased from Peptides International (Louisville, KY). LPS was obtained from Sigma-Aldrich (Cat #L4391, *Escherichia coli* 0111:B4). Fluorophore-conjugated monoclonal antibodies for flow cytometry were obtained from BD Biosciences (CD11b-PeCy7 or APC-Cy7, CD45-PerCP or PE-Cy7, ICAM1-APC, CD69-PECy7, pS727-STAT1-Alexa647), eBiosciences (CD3-FITC, CD8-APC, CD95-PE) and Cell Signaling Technologies (pY701-STAT1-Alexa488).

### Primary microglia isolation and cell culture

Adult (8–12-week-old) female C57BL/6 mice were euthanized, and the brains were isolated following rapid cold saline perfusion. Single cell suspensions from brain homogenates were washed followed by Percoll-gradient centrifugation and isolation of the mononuclear cell fraction as previously described [13]. Brain mononuclear cells (76–85% pure) were used for flow cytometric studies and for in vitro CD8^+^ T cell proliferation experiments. BV2 microglia were maintained in Dulbecco’s modified Eagle Medium (with 10% FBS).

### In vivo studies

Mice were housed in the Department of Animal Resources at Emory University under standard conditions, no special food/water accommodations. Institutional Animal Care and Use Committee approval was obtained prior to in vivo work. Adult (8–12-week-old) female C57BL/6 mice were treated with four once daily intraperitoneal doses of sterile PBS, ShK-223 (100 μg/kg), LPS (20 μg), or LPS+ShK-223 [14]. Mice were then anesthetized with isoflurane and rapidly cardiac-perfused with cold PBS over 2 min, after which microglia were isolated for flow studies or for in vitro T cell proliferation assays.

### Gap closure assay

BV2 microglia were grown to near confluence in 12 well plates followed by exposure to PBS, ShK-223 (100 nM), LPS (100 ng/mL), or LPS+ShK-223. Two para-median scratches were placed in each well and imaged using an inverted microscope at time 0 and at 24 h and areas of each gap at each time point were calculated (ImageJ), and relative gap closure over 24 h was determined (*n* = 6 biological replicates per condition).

### Transmigration assay

Fifty thousand BV2 microglia grown in serum-free DMEM were added to the upper chamber of fibronectin-coated transwell chambers (8-μm pore size, Costar Sigma-Aldrich #3464) in the presence of either PBS, ShK-223 (100 nM), LPS (100 ng/mL), or LPS+ShK-223. Separate experiments were performed to test the effects of 100 nM ShK-186, a well-characterized selective Kv1.3 channel blocker, on LPS-induced effects on transmigration. Cells were allowed to transmigrate over 24 h into the lower chamber containing either serum-free medium (lacking chemotactic factors) or medium supplemented with 10% FBS (to induce chemotaxis). Cytochalasin D (50 μM) was also added to the upper chamber in a separate experiment to inhibit actin-dependent transmigration into serum-containing medium. All experiments were carried out in biological triplicates.

### Flow cytometry studies

Flow cytometric analyses of BV2 microglia and freshly isolated primary mouse microglia as well as T cells from T cell proliferation assays were performed using well-characterized fluorophore-conjugated monoclonal antibodies against CD11b, CD45, ICAM1, CD69, MHCI (H2Kb), CD3, and CD8. Functional cell surface Kv1.3 channels expressed by BV2 or primary microglia were assessed using ShK-F6CA labeling via a previously validated assay [15]. For primary mouse microglia studies, gating strategies were employed to differentiate resident microglia (CD11b^+^ CD45^low^) from peripherally derived brain-infiltrating macrophages (CD11b^+^ CD45^high^) [16]. Phospho-flow cytometry was performed to detect pS727 and pY701 STAT1 phosphorylation (pY701-Alexa488 ab: Cell Signaling, pS727-Alexa647: BD Biosciences) [17]. EHD1 was detected in separate experiments using anti-EHD1 rabbit polyclonal Ab (1:100 [Abcam Cat#EPR4954]) followed by 1:500 Anti-rabbit DyLight-549 (Jackson Laboratories) after cells were fixed (Fixation buffer, eBioscience #00822249) and permeabilized (Permeabilization buffer eBioscience #00833356). Blocking with 10% horse serum and appropriate control experiments (unstained cells, cells treated with secondary Ab only) were performed. Compensation experiments were performed prior to each experiment using compensation beads (OneComp eBeads Thermo-Fisher Sci. #01111141). Samples were analyzed on a BD FACSCanto II flow cytometer, and data were collected with CellQuest software and analyzed with FlowJo software (Version 10).

### Phagocytosis assay

The ability of BV2 microglia and freshly isolated primary microglia to phagocytose red-fluorescent polystyrene 1-μm microspheres (Thermo-Fisher FluoroSpheres Cat#F13083) was assessed. Cells were exposed to 2.5 μL of the beads (≈200 beads per cell) for 30 min at 37 ° C, followed by washing and flow cytometry [18]. Fluorescence of single microspheres was determined by running beads in isolation and proportion of cells that phagocytosed at least one bead was determined and compared across the treatment groups. BV2 cells were exposed to PBS, LPS, ShK-223, or LPS+ShK-223. Similarly, primary microglia were isolated from the brains of mice treated with four daily IP doses of PBS, LPS, ShK-223, or LPS+ShK-223, followed by flow cytometric analysis after labeling with anti-CD11b and anti-CD45 fluorophore-conjugated antibodies.

### Reactive oxygen species assay

Freshly isolated brain mononuclear cells from mice (treated with PBS, LPS, ShK-223 or LPS+ShK-223) were incubated with 5 μM dichlorofluorescein diacetate (DCFDA, Thermo-Fisher #D399) for 30 min, washed in PBS and then analyzed by flow cytometry. DCFDA is a cell-permeant dye that is de-acetylated by cellular esterases to a non-fluorescent compound which is then oxidized by hydroxyl, peroxyl, and other reactive oxygen species (ROS) to a green fluorescent compound that can be measured by fluorescence microscopy or flow cytometry.

### Immunofluorescence microscopy

BV2 cells were grown on glass coverslips and exposed to LPS, ShK-223, or LPS+ShK-223 for 24 h, washed twice with PBS and then treated with ShK-F6CA (100 nM) for 30 min, then fixed using 4% formaldehyde for 15 min. In separate experiments, BV2 cells were incubated with anti-EHD1 rabbit polyclonal antibody (Abcam Cat#EPR4954) as well. Following fixation, the cells were washed and permeabilized with 2 mL 0.1% Triton X-100 in PBS for 15 min on ice. Cells were washed thrice followed by image acquisition on an immunofluorescence microscope (Microscope: Olympus BX51 and camera: Olympus DP70). Cells were also incubated with anti-CD14-PE antibody and co-localization between ShK-F6CA and anti-CD14 labeling was determined by immunofluorescence microscopy and image processing using ImageJ software.

### Mass spectrometry sample preparation

Samples were prepared essentially as described (CITE CELLS SYSTMES) with slight modifications [19]. BV2 microglia were grown to 75% confluence and then exposed to PBS, LPS (100 ng/mL), ShK-223 (100 nM), or LPS+ShK-223 for 24 h and then harvested. Each cell pellet was individually homogenized in 300 μL of urea lysis buffer (8 M urea, 100 mM NaHPO_4_, pH 8.5), including 3 μL (100× stock) HALT protease and phosphatase inhibitor cocktail (Pierce). All homogenization was performed using a Bullet Blender (Next Advance) according to manufacturer protocols and as previously published [20]. Briefly, each cell pellet was added to Urea lysis buffer in a 1.5-mL Rino tube (Next Advance) harboring 750 mg stainless steel beads (0.9–2 mm in diameter) and blended twice for 5-min intervals in the cold room (4 °C). Protein supernatants were transferred to 1.5-mL Eppendorf tubes and sonicated (Sonic Dismembrator, Fisher Scientific) three times for 5 s with 15 s intervals of rest at 30% amplitude to disrupt nucleic acids and subsequently vortexed. Protein concentration was determined by the bicinchoninic acid (BCA) method, and samples were frozen in aliquots at −80 °C. Protein homogenates (100 μg) were diluted with 50 mM NH_4_HCO_3_ to a final concentration of less than 2 M urea and then treated with 1 mM dithiothreitol (DTT) at 25 °C for 30 min, followed by 5 mM iodoacetimide (IAA) at 25 °C for 30 min in the dark. Protein was digested with 1:100 (*w*/*w*) lysyl endopeptidase (Wako) at 25 °C for 2 h and further digested overnight with 1:50 (*w*/*w*) trypsin (Promega) at 25 °C. Resulting peptides were desalted with a Sep-Pak C18 column (Waters) and dried under vacuum. For LC-MS/MS analysis, derived peptides were re-suspended in 100 μL of loading buffer (0.1% formic acid, 0.03% trifluoroacetic acid, 1% acetonitrile). Peptide mixtures (2 μL) were separated on a self-packed C18 (1.9 μm, Dr. Maisch, Germany) fused silica column (25 cm × 75 μM internal diameter (ID); New Objective, Woburn, MA) by a Dionex Ultimate 3000 RSLCNano and monitored on a Fusion mass spectrometer (Thermo-Fisher Scientific, San Jose, CA). Elution was performed over a 2 h gradient at a rate of 400 nL/min with buffer B ranging from 3 to 80% (buffer A: 0.1% formic acid in water, buffer B: 0.1% formic acid in acetonitrile). The mass spectrometer cycle was programmed to collect at the top speed for 3-s cycles. The MS scans (400–1600 m/z range; 200,000 AGC; 50 ms maximum ion time) were collected at a resolution of 120,000 at 200 m/z in profile mode and the HCD MS/MS spectra (0.7 m/z isolation width; 30% collision energy; 10,000 AGC target; 35 ms maximum ion time) were detected in the ion trap. Dynamic exclusion was set to exclude previous sequenced precursor ions for 20 s within a 10 ppm window. Precursor ions with +1 and +8 or higher charge states were excluded from sequencing.

### MaxQuant for label-free quantification and data analysis

Raw data files were analyzed using MaxQuant v1.5.2.8 with Thermo Foundation 2.0 for RAW file reading capability, as previously published [20]. The search engine Andromeda was used to build and search a concatenated target-decoy IPI/Uniprot mouse reference (downloaded Aug 14, 2015). Protein methionine oxidation (+15.9949 Da) and protein N-terminal acetylation (+42.0106 Da) were variable modifications (up to five allowed per peptide); cysteine was assigned a fixed carbamidomethyl modification (+57.0215 Da). Only fully tryptic peptides were considered with up to two miscleavages in the database search. A precursor mass tolerance of ±10 ppm was applied prior to mass accuracy calibration and ±4.5 ppm after internal MaxQuant calibration. Other search settings included a maximum peptide mass of 6000 Da, a minimum peptide length of six residues, and 0.6 Da Tolerance for ion trap (IT) HCD MS/MS scans. Co-fragmented peptide search was enabled to deconvolute multiplex spectra. The false discovery rate (FDR) for peptide spectral matches, proteins, and site decoy fraction were all set to 1%. Quantification settings were as follows: re-quantify with a second peak finding attempt after protein identification has completed; match full MS1 peaks between runs; a 1.5-min retention time match window was used after an alignment function was found with a 20 min RT search space. The label-free quantitation (LFQ) algorithm in MaxQuant [21, 22] was used for protein quantitation. Volcano plots were plotted with ggplot2 packages in R. Proteins with >25% overall missing data or more than one missing data point per treatment group were excluded from analysis.

### Statistical and gene ontology pathway analysis

One-way ANOVA (*p* ≤ 0.05) was used as the first threshold for differences across treatment groups. Then, a two-tailed Student’s *t* test was performed comparing the LPS and control groups and proteins that showed statistically significant (*p* ≤ 0.05), and at least 1.25-fold change in both directions were identified. Within these LPS-regulated proteins, pairwise comparisons between LPS- and LPS+ShK-223-treated groups were performed (threshold *p* ≤ 0.05). A less-stringent approach (*p* value ≤0.10) for all comparisons described was also taken. Proteins which had expression altered by ShK-223 treatment without LPS were also identified by performing pairwise comparisons between the control and ShK-223 groups (*p* ≤ 0.05 and at least 1.25 fold change in either direction). Gene ontology (GO) analyses were performed using the following lists of proteins (list 1: All LPS-regulated proteins with reversal by ShK-223; list 2: LPS-upregulated proteins with reversal by ShK, list 3: LPS-downregulated proteins with reversal by ShK, list 4: ShK-upregulated without LPS, and list 5: ShK-downregulated without LPS) with the background of all sequenced mouse proteins in the entire dataset (3141 proteins). Functional enrichment was determined using the GO-Elite (v1.2.5) package [20, 23]. The set of total proteins identified was used as the background. Z-score determines overrepresentation of ontologies in a module, and permutation *P* value was used to assess the significance of the Z-score. For these significantly changed proteins, a Z-score cutoff of 1.96, with *p* value cutoff of 0.05 with a minimum of three proteins per category were used as filters in pruning the ontologies. Circle plots were plotted with circlize package in R software and exploratory pathway analyses were performed using the most highly enriched GO terms in each list using canonical pathway analysis in Metacore (Thomson-Reuters) [24]*.* This manually curated database captures high-quality experimental evidence from peer-reviewed literature and through curating interactions, omics-disease relationships, and constructing canonical pathways from these omics experiments and allows reduction of high dimensional data into a systems biology context.

### OT-1 CD8^+^ T cell proliferation assay by CFSE dilution

Ovalbumin (Ova 257-264)-specific CD8-positive T cells were isolated from the spleen of adult OT-1 mice using standard protocols for T cell enrichment (EasySep Kit #19751, Stemcell Tech). T cells were labeled with CFSE (CellTrace™ Cat# C34554) and 100,000 cells were co-cultured with 20,000 Ova-loaded microglia (2 μg/mL for 30 min, Sigma Cat#S7951) that were derived from either (i) BV2 microglia pre-treated with PBS, LPS, ShK-223, or LPS+ShK-223 for 24 h or (ii) primary microglia freshly isolated from the brains of mice that had been treated with four daily IP doses of PBS, LPS, ShK-223, or LPS+ShK-223 as described. After 48 h, T cells were harvested and assessed for CFSE dilution by flow cytometry [18].

### Reverse transcriptase quantitative PCR

Real-time PCR was performed as previously reported [25]. Total RNA was isolated from cells using a standard Trizol (Invitrogen) extraction protocol using an RNeasy mini extraction kit (Qiagen) according to the manufacturer’s instructions. RNA was converted to cDNA using a high-capacity cDNA reverse transcription kit (Ambion). Real-time PCR was performed on a 7500 Fast RT-PCR instrument (Applied Biosystems) using 20 ng/uL cDNA, TaqMan PCR master mix (Applied Biosystems), and gene-specific TaqMan probes (Applied Biosystems) against TAP1 (Mm00443188_m1), EHD1 (Mm01236839_m1), IRF1 (Mm01288580_m1), IRF7 (Mm00516793_g1), NFkB (Mm00476361_m1), IL1B (Mm00434228_m1), Arg1 (Mm00475988_m1), GAPDH (Mm99999915_g1), and HPRT (Mm03024075_m1). For each RNA sample, each primer set was run in triplicate. Gene expression was normalized to the internal control GAPDH (for BV2 microglia) or HPRT (for primary microglial cells), and relative expression was calculated for each gene using the 2ΔΔCT method after normalizing to the control sample.

### Western blot experiments

Western blot studies were performed per our standard protocols [7]. After overnight protein transfer, PVDF membranes were blocked with 5% fat-free milk for 1 h followed by incubation with primary antibodies overnight. The immunoblots were incubated with anti-Kv1.3 rabbit polyclonal antibody (APC 101, Alomone labs, 1:1000) and anti-beta-actin mAb (Cell Signaling, #3873,1:1000) for 24 h and subsequently appropriate fluorescent secondary antibodies (anti-mouse IgG IRDye® 800 conjugate, Rockland, 1:20,000 and anti-rabbit IgG Alexa Fluor® 680 conjugate, Invitrogen) were added for 1 h. An Odyssey Scanner (LI-COR) was used to visualize labeled proteins.

### Other statistical considerations

Graphs were created using Graphpad Prism Version 5 or SPSS Version 22. Data are shown as mean ± SEM. For group-wise comparisons, one-way ANOVA was performed to detect differences across groups and post hoc pairwise comparisons were performed using Tukey’s HSD test. Paired analyses were performed for pairwise comparisons of normalized qRT-PCR data. Statistical significance was set at *p* value ≤0.05 for all experiments unless separately specified.

## Results

### Kv1.3 channel upregulation by microglia and brain-infiltrating macrophages following LPS activation

**Fig. 1:**
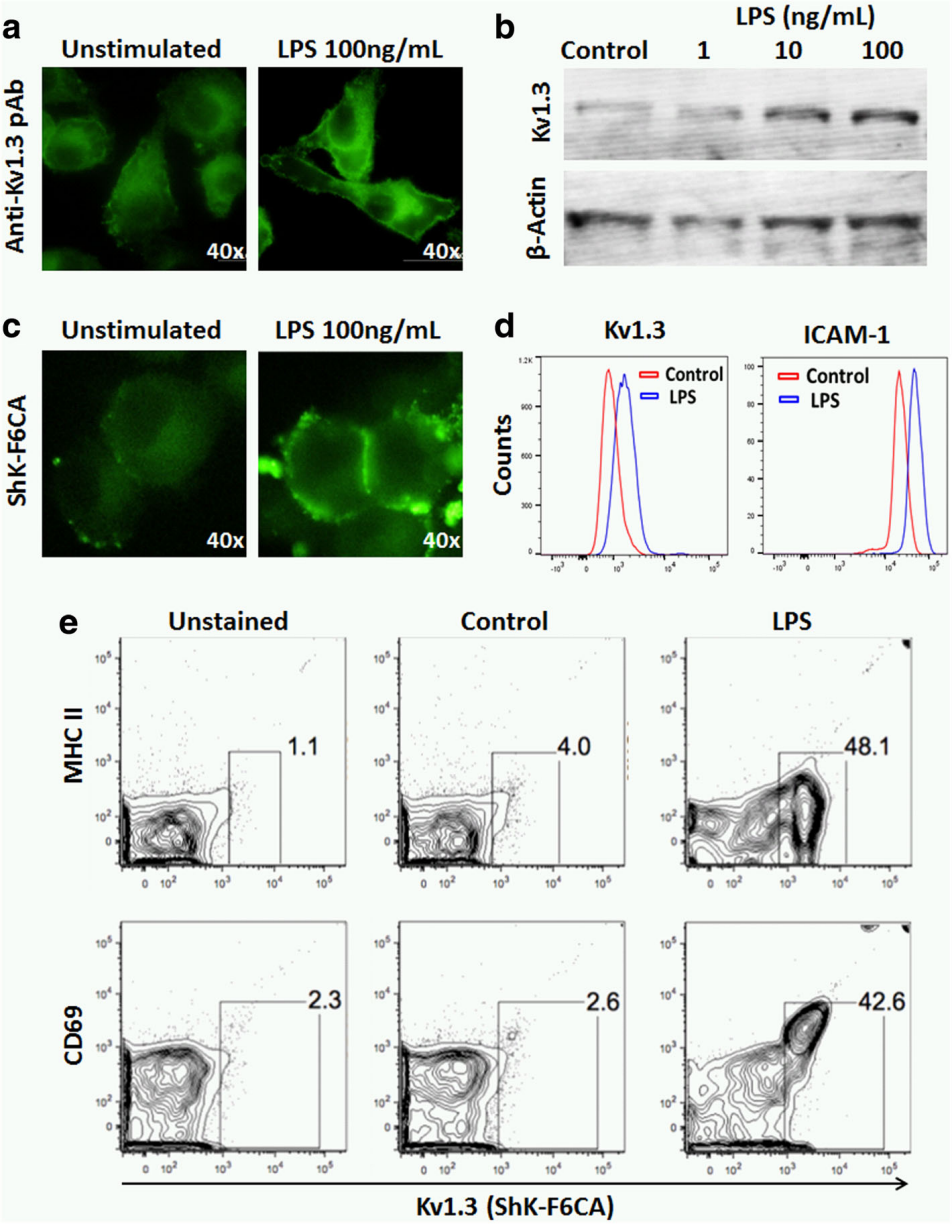
LPS-activated BV2 microglia upregulate Kv1.3 channels. **a** Immunofluorescence microscopic comparison of Kv1.3 protein expression (detected by Anti-Kv1.3 rabbit pAb 1:500) by unstimulated and LPS-activated (100 ng/mL × 24 h) BV2 microglia. **b** Western blot confirmation of LPS dose-dependent increase in Kv1.3 protein in BV2 whole-cell lysates (Anti-Kv1.3 pAb 1:1000). **c** Immunofluorescence microscopic comparison of cell surface Kv1.3 channels in unstimulated and LPS-activated BV2 cells using ShK-F6CA. **d** Flow cytometric comparison of ShK-F6CA-labeled cell surface Kv1.3 channels and microglial activation marker ICAM-1 in unstimulated and LPS-activated BV2 cells. **e** Assessment of microglial activation markers MHCII and CD69 in unstimulated and LPS-activated BV2 cells

LPS, a bacterial endotoxin, is a prototypic pro-inflammatory activator of microglia and macrophages that has pleiotropic systemic and neuro-inflammatory effects that recapitulate chronic inflammation in neurodegenerative diseases [26, 27]. We first investigated whether Kv1.3 channels are upregulated following LPS-induced pro-inflammatory activation of microglia, using both in vitro and in vivo models. BV2 microglia were stimulated in vitro with LPS (100 ng/mL) for 24 h to mimic pro-inflammatory activation. We found that LPS-upregulated Kv1.3 channel protein expression by BV2 microglia by immunofluorescence microscopy (Fig. 1a) and observed an LPS dose-dependent increase in Kv1.3 protein by immunoblotting (Fig. 1b). We then utilized a previously validated fluorescent assay for functional and Kv1.3 channel detection that uses a green fluoresceinated ShK analog (ShK-F6CA) that binds to cell surface Kv1.3 channels with high selectivity and affinity (IC_50_ = 49 pM) at 1:1 stoichiometry [15]. ICAM-1, an activation marker of microglia, was also used to confirm LPS-induced activation. As expected, ShK-F6CA labeling of cell surface Kv1.3 channels was increased after LPS activation as compared to resting BV2 microglia in immunofluorescence (Fig. 1c) and flow cytometric experiments (Fig. 1d). ShK-F6CA labeling of Kv1.3 channels could be blocked by competition via pre-incubation with saturating concentrations of non-fluorescent Kv1.3-blocking ShK analog ShK-223 (Additional file 1: Figure S1). We also confirmed LPS-induced pro-inflammatory activation of BV2 microglia as demonstrated by higher surface ICAM1, MHCII, and CD69 expression (Fig. 1d–e) [28].

**Fig. 2:**
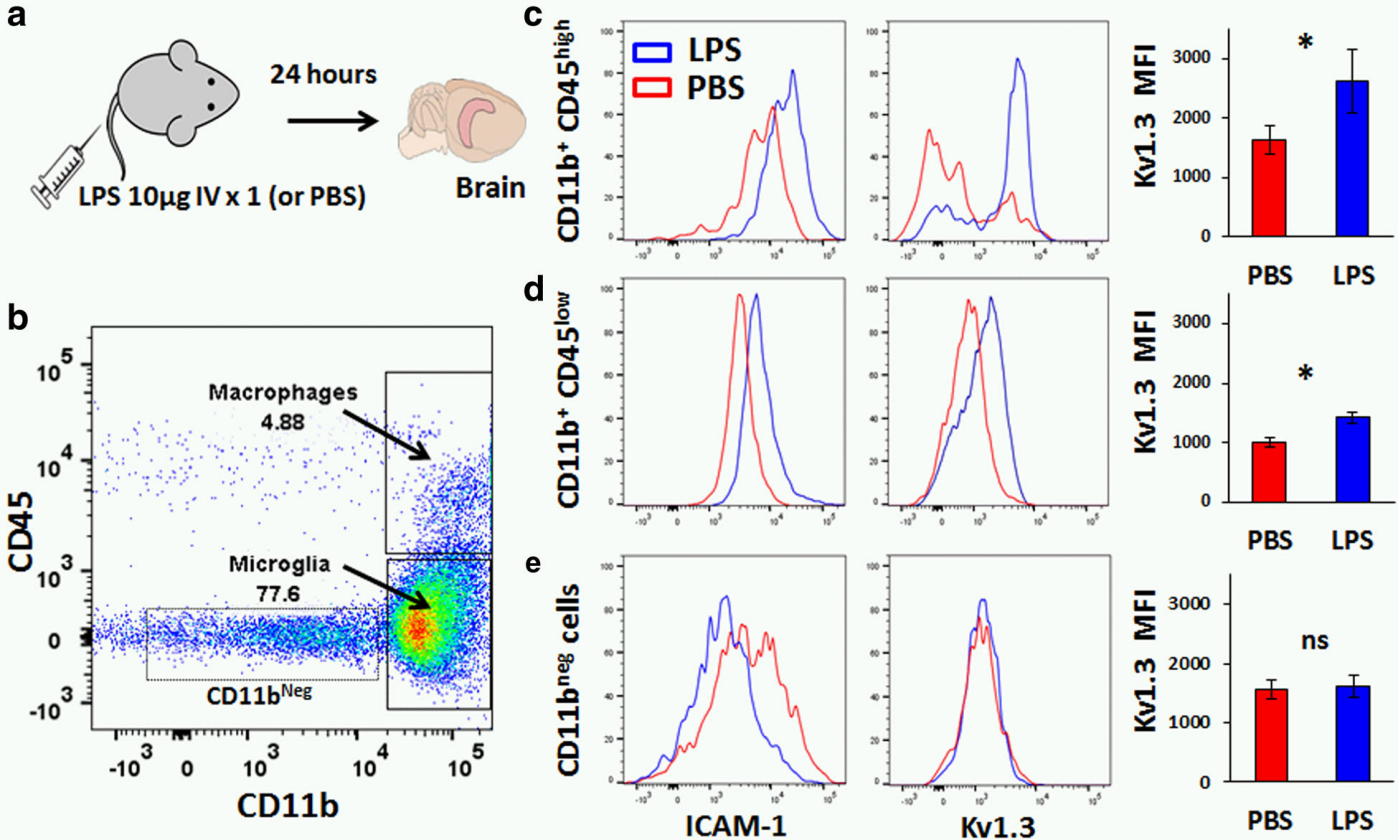
In vivo demonstration of Kv1.3 channel upregulation by microglia and brain-infiltrating macrophages in a mouse model of LPS-induced neuroinflammation. **a** LPS was administered to adult C57BL/6 mice by tail vein injection, and brain mononuclear cells were analyzed by flow cytometry (CD11b, CD45, ShK-F6CA, and ICAM-1). **b** Most cells were CD11b^+^CD45^low^ microglia while small populations of brain-infiltrating macrophages CD11b^+^CD45^high^ and CD11b^-^ non-myeloid cells were observed. **c**–**e** Comparison of Kv1.3 channel and ICAM-1 expression in brain mononuclear cells isolated from control and LPS-treated mice in microglia (**c**), brain-infiltrating macrophages (**d**), and CD11b^-^ subpopulations (**e**) (*p < 0.05, **p < 0.01, ***p < 0.005)

BV2 microglia are a suitable model to study pro-inflammatory LPS activation but significant differences between BV2 and primary microglia have been observed, and as a result, observations using BV2 microglia need to be validated using primary microglia [29, 30]. To better mimic in vivo microglial activation, we administered LPS to adult wild-type mice (single 10-μg injection by tail vein, Fig. 2a) [14]. Brain mononuclear cells were isolated from the brain and labeled for CD11b, CD45, ICAM-1, and ShK-F6CA. Microglia (CD11b^+^CD45^low^) accounted for the majority of isolated cells while brain-infiltrating macrophages (CD11b^+^CD45^high^) accounted for a minority (Fig. 2b). As compared to PBS-treated animals, microglia (Fig. 2d) and macrophages (Fig. 2e) isolated from the brains of LPS-treated animals expressed higher levels of ICAM-1 and ShK-F6CA labeling. Interestingly, baseline level of Kv1.3 channel expression was also higher in CD45^high^ brain-infiltrating macrophages as compared to that in CD45^low^ microglia and the magnitude of Kv1.3 channel upregulation by LPS was also highest in the CD45^high^ macrophage population (Fig. 2c-d). CD11b^−^ cells, in contrast to CD11b^+^ cells, did not exhibit changes in ICAM1 or Kv1.3 channel expression (Fig. 2e). LPS-treated mice also had a greater proportion of macrophages in the brain, indicating increased steady state levels, trafficking, or blood–brain barrier permeability induced by systemic inflammation. As compared to brain-infiltrating macrophages, Kv1.3 channel expression was found to be very low in splenic macrophages (Additional file 1: Figure S2). These data demonstrate upregulation of Kv1.3 channels by microglia and brain-infiltrating macrophages following LPS activation, in both microglial cell line and in vivo model of LPS activation.

### Quantitative proteomics reveals Kv1.3-dependent processes in pro-inflammatory and resting microglia

**Fig. 3:**
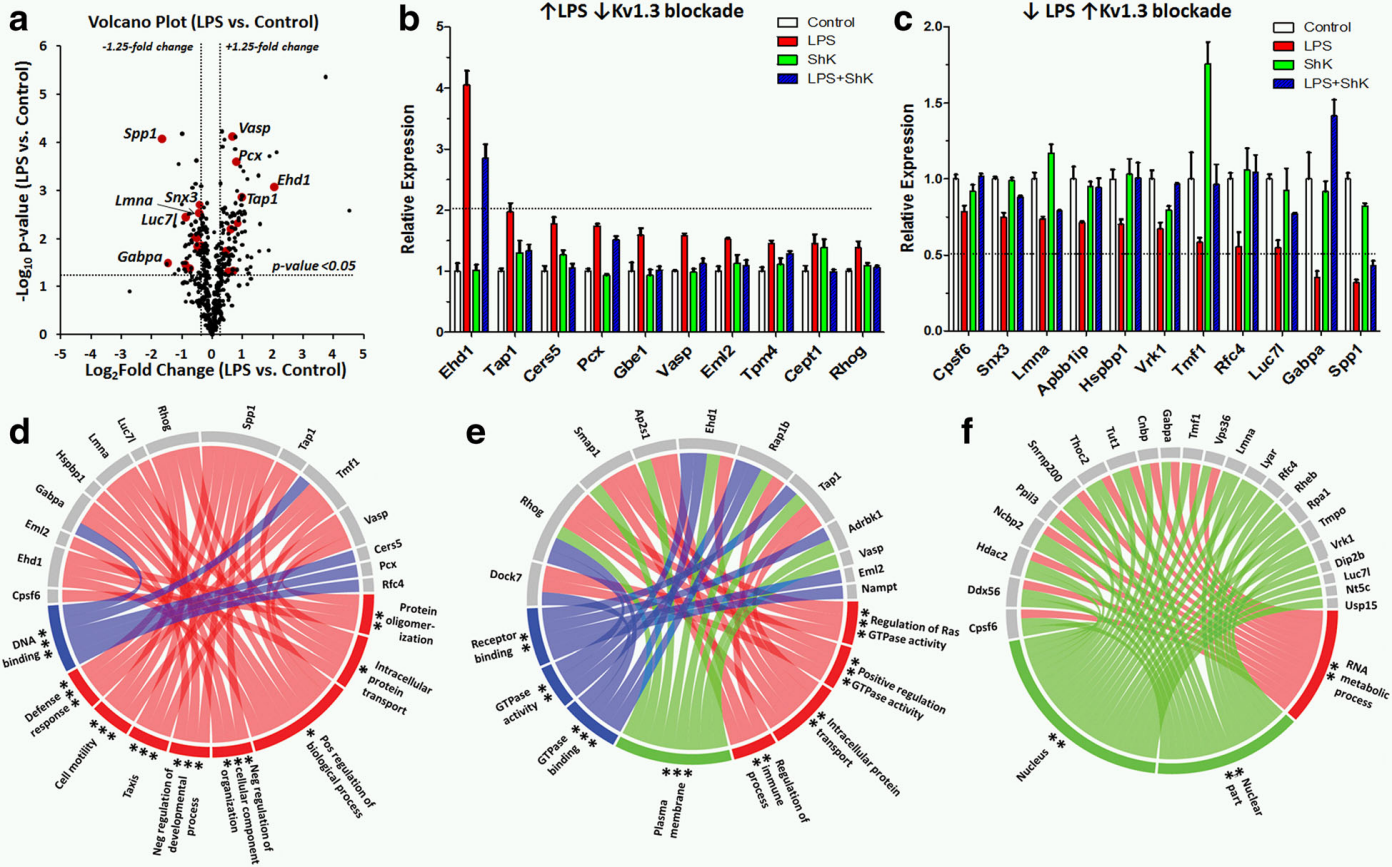
Identification of novel Kv1.3 channel-dependent molecular mechanisms by microglial quantitative proteomics. BV2 microglia were exposed to PBS, LPS (100 ng/mL), ShK-223 (100 nM), or LPS+ShK-223 for 24 h (3 replicates/group) and whole-cell lysates were used for mass-spectrometric analysis. **a** Volcano-plot: Of 450 proteins differentially expressed across all 4 groups (*black dots*), 144 proteins were significantly up- or downregulated following LPS exposure (*top-right* and *top-left* quadrants; *vertical dotted lines* represent the upper and lower boundaries of 1.25-fold change threshold while the *horizontal dotted line represents p* value threshold of 0.05 comparing LPS vs. control groups). Among these, ShK-223 reversed the LPS effect in 21 proteins (highlighted in *red*). **b** LPS-upregulated proteins that were reversed by ShK-223 and **c** LPS-downregulated proteins reversed by ShK-223 are shown. Relative expression represents label-free quantitation data in each treatment condition normalized to the control PBS group. The *dotted horizontal line* represents the twofold change threshold as compared to the PBS group. **d**–**f** *Circle plots* representing results from GO analyses are shown (*gray*: protein/gene symbol, *red*: biological process, *green*: cellular component, *purple*: molecular function). Only significantly enriched GO terms (unadjusted *p* ≤ 0.05 based on enrichment score) are shown (*p < 0.05, **p < 0.01, ***p < 0.005). **d** GO analysis of 21 LPS-regulated Kv1.3-dependent proteins (*n* = 21, stringent *p* ≤ 0.05 threshold comparing LPS vs. LPS+ShK-223). **e** GO analysis of LPS-upregulated Kv1.3-dependent proteins (*n* = 26, *p* ≤ 0.10 threshold comparing LPS vs. LPS+ShK-223). **f** GO analysis of LPS-downregulated Kv1.3-dependent proteins (*n* = 40, *p* ≤ 0.10 threshold comparing LPS vs. LPS+ShK-223)

Our data show that upregulation of Kv1.3 channels parallels pro-inflammatory LPS-induced microglial activation in BV2 as well as primary mouse microglia. Potassium channels regulate membrane potential and calcium signaling in immune cells and, through their co-assembly with key signaling complexes during immune activation, regulate specific signaling cascades that in turn impact gene transcription and cellular processes [31]. To investigate the molecular mechanisms in pro-inflammatory microglial activation that are regulated by Kv1.3 channels, we performed unbiased proteomic screens of unstimulated and LPS-activated BV2 microglia, in the presence or absence of a potent Kv1.3 channel blocker ShK-223 (100 nM). As expected, LPS activation (100 ng/mL) induced clear morphological differences at 24 h (Additional file 1: Figure S3). No changes in cell viability were noted across treatment conditions. At the 24-h time point, we identified 3141 proteins across 12 proteomic datasets (Control, ShK-223, LPS, LPS+ShK-223; *n* = 3/group) of which 81.6% proteins had no missing data (Additional file 1: Figure S4). Of these 3141 proteins (Additional file 2: Table S1-S2), 450 were differentially expressed across the four groups (ANOVA *p* value ≤0.05). Of these 450 proteins (Fig. 3a), 144 were either significantly up- or downregulated by LPS stimulation (fold change ≥1.25 and pairwise *t* test *p* ≤0.05 comparing LPS vs. control). This list was also consistent with previously published proteomic data (35.9% agreement) and transcriptomic data (94.4% agreement) from LPS-treated mouse microglia (Additional file 2: Table S3) [32, 33]. As predicted, GO analysis of the 144 LPS-regulated proteins confirmed that LPS activation resulted in enrichment of pro-inflammatory biological functions, cell proliferation, and immune activation and qRT-PCR confirmed upregulation of pro-inflammatory genes IL1B, IRF1, and IRF7 and down regulation of Arg1 (Additional file 1: Figure S5). Top proteins that were downregulated by LPS treatment included known anti-inflammatory microglial proteins (ARG1), DNAJC21, LUC7L, and transcription factor Jun (see Additional file 2: Table S1 for complete list). Among the 144 LPS-regulated proteins, 21 showed statistically significant reversal (*p* ≤ 0.05) of the LPS effect by ShK-223 (Fig. 3a–c). Ten of these were proteins that were upregulated by LPS (Fig. 3b) while 11 were proteins downregulated by LPS (Fig. 3c). EHD1 and TAP1 proteins demonstrated the highest upregulation while SPP1 and GABPA showed the most downregulation following LPS exposure. We confirmed by immunofluorescence microscopy that EHD1 protein expression was increased by LPS, and this effect was reversed by ShK-223 treatment (Additional file 1: Figure S6). However, we did not observe an effect of ShK-223 on LPS-induced TAP1 and EHD1 transcription in BV2 microglia by qRT-PCR. A combined GO analysis of these 21 proteins revealed enrichment of several biological process terms including “taxis,” “cell motility” and “positive and negative regulation of biological processes,” “defense response,” and “protein oligomerization” (Fig. 3d) suggesting that Kv1.3 channels may regulate cell motility and taxis in LPS-activated microglia. Using less-stringent criteria (*p* ≤ 0.1 comparing LPS vs. LPS+ShK-223), an additional 46 LPS-regulated and Kv1.3-dependent proteins were identified. Among the LPS-upregulated Kv1.3 channel-dependent proteins (*n* = 27), the top enriched GO terms (Fig. 3e) were biological process terms “regulation of GTPase activity,” “immune process regulation,” and “intracellular protein transport (Fig. 3e).” Several of these proteins were localized to plasma membrane. GO analysis of LPS-downregulated Kv1.3-dependent proteins (*n* = 40) showed enrichment of the biological process term “RNA metabolic process” and were localized to the nucleus suggesting a novel role for Kv1.3 channels in regulating nuclear and RNA processes (Fig. 3f). We also identified significant effects of Kv1.3 channel blockade on 53 proteins (≥1.25 fold change and *p* ≤ 0.05) in unstimulated BV2 microglia (24 upregulated and 29 downregulated by ShK-223, Additional file 2: Table S4) of which two proteins (TMF1 and VRK1) were common to those also altered by LPS activation. GO analyses showed enrichment of cellular component term “organelle membrane” for proteins upregulated by ShK-223 and of biological process term “cellular macromolecule catabolic process” for proteins downregulated by ShK-223. Our results identify novel Kv1.3 channel-dependent proteins and predict regulatory roles for Kv1.3 channels in microglial taxis, focal adhesion formation, antigen and intracellular protein transport, and RNA processing.

### Kv1.3 channels regulate taxis and focal adhesion formation in LPS-activated microglia

**Fig. 4:**
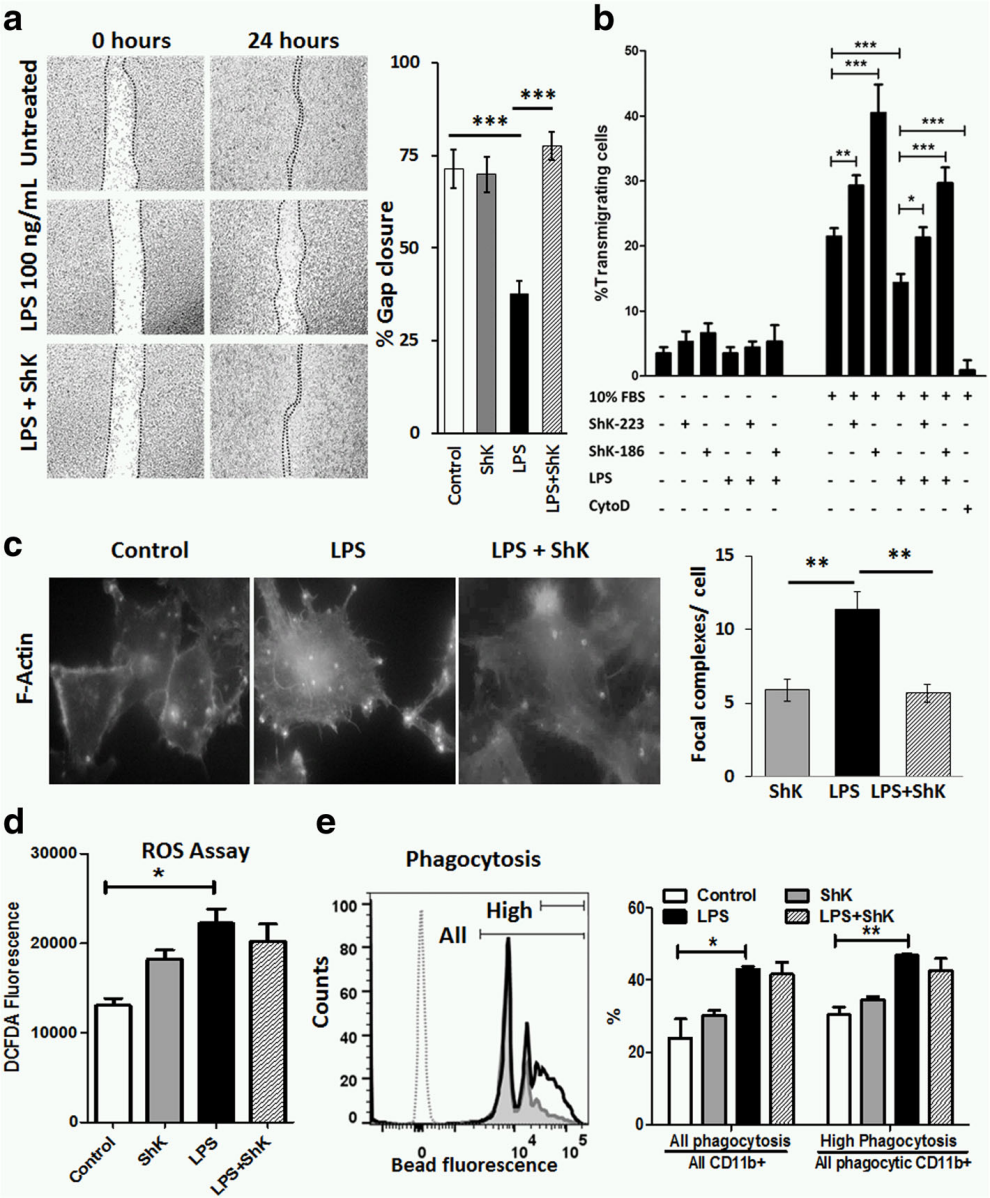
Kv1.3 channels regulate microglial taxis and formation of F-actin complexes induced by LPS. **a** In the gap closure assay of microglial taxis, BV2 cells were grown to near confluence followed by placement of a uniform scratch using a 200-μm pipette tip. Ability of microglia to close this gap was assessed by measuring the percentage of gap closure over a 24-h period. Paired (0 and 24 h) representative images from each treatment group are shown (**a**, *left*), and the comparison of percentage gap closure (over 24 h) is shown (**a**, *right*) (six replicates per condition). **b** BV2 microglial transmigration across a transwell membrane (8-μm pore diameter) after exposure to control, ShK-223, ShK-186, LPS or LPS+ShK-223, or ShK-186, towards serum-containing medium (10% fetal bovine serum). Following 24 h of transmigration, cells were detached from undersurface of the insert (0.25% Trypsin), and cells that successfully migrated across the membrane were counted on a hemocytometer (*n* = 3, independent experiments). **c** Comparison of F-actin containing focal adhesion complexes in BV2 microglia following exposure to control, ShK-223, LPS, or LPS+ShK-223. Fixed and permeabilized BV2 cells were labeled with phalloidin-rhodamine to detect F-actin (*left*: immunofluorescence images). The number of focal complexes were counted per cell at ×40 magnification (*right*) and compared (>25 cells counted per condition). **d** DCFDA assay of ROS production by brain mononuclear cells isolated from C57BL/6 mice treated with PBS, LPS, ShK-223, or LPS+ShK-223 IP for four consecutive days (*n* = 3, mice/group). Cells were loaded with DCFDA for 30 min and assayed for ROS activity by flow cytometry. **e** Flow cytometric phagocytosis assay of fluorescent (PE) microbeads by brain mononuclear cells isolated from C57B6/L mice treated with PBS, LPS, ShK-223, or LPS+ShK-223, *n* = 3/group). *Dotted line*: fluorescence of cells not exposed to beads; *gray histogram*: PBS-treated; *black histogram*: LPS-treated. The proportions of all phagocytic cells and highly phagocytic cells were compared across treatment groups (*p < 0.05, **p < 0.01, ***p < 0.005)

GO analyses of our proteomic data suggest a role for Kv1.3 channels in cell adhesion and taxis in LPS-activated microglia. Therefore, we hypothesized that Kv1.3 channels regulate LPS-induced migratory responses to local tissue injury and regulate formation of focal adhesion complexes in BV2 microglia. In the gap closure assay of taxis, microglia respond to a local tissue injury (scratch) by chemotaxis and proliferation to close the gap within 24 h. This homeostatic function was inhibited by LPS, presumably due to increased adhesiveness of activated microglia. While ShK-223 had no direct effect on gap closure, ShK-223 pre-incubation partially reversed the inhibitory effect of LPS (Fig. 4a). In the transmigration assay, LPS inhibited migration of BV2 cells from the upper chamber towards serum-containing medium in the lower chamber while Kv1.3 blockade by ShK-223 rectified this inhibition. As compared to control, ShK-223 also augmented transmigration towards serum. Minimal migration of BV2 cells was observed in the absence of serum in the lower chamber (Fig. 4b). ShK-186, a well-characterized and highly selective inhibitor of Kv1.3 channels, also demonstrated similar effects on transmigration (Fig. 4b). Kv1.3 channel blockade also decreased LPS-induced increase in F-actin-enriched focal adhesion complexes in BV2 microglia (Fig. 4c). Unlike gap closure and focal adhesion complex formation in microglia, we found in in vivo experiments that LPS-induced reactive oxygen species production measured by the DCFDA assay (Fig. 4d) and LPS-augmented phagocytosis of red-fluorescent polystyrene microspheres (Fig. 4e) in brain mononuclear cells were not affected by Kv1.3 channel blockade. These results validate predictions based on our proteomic data and confirm that Kv1.3 channels regulate LPS-induced focal adhesion formation as well as inhibition of migration.

### Kv1.3 channels regulate EHD1, TAP1, GABPA, and pro-inflammatory gene expression in primary mouse microglia

**Fig. 5:**
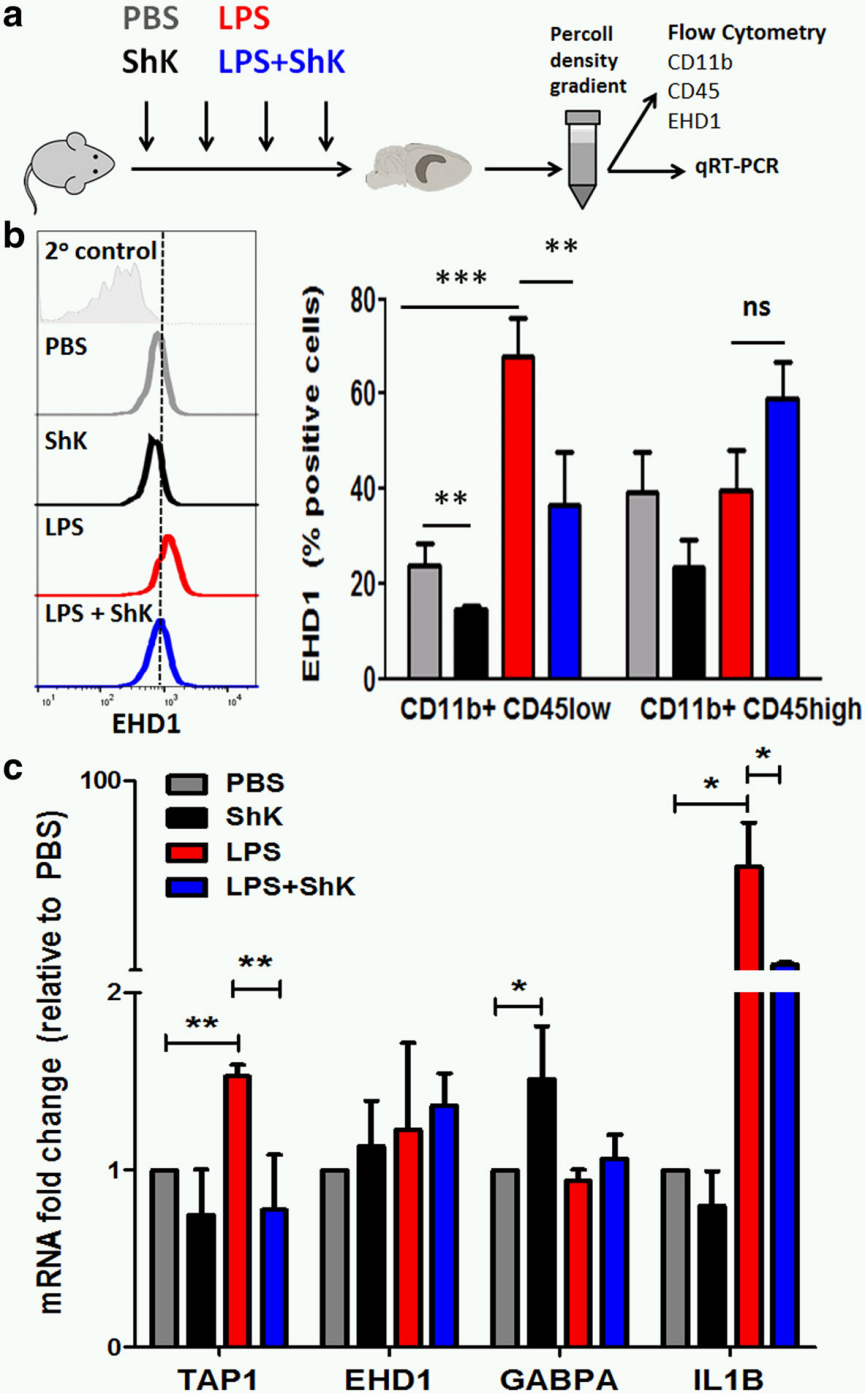
Kv1.3 channels regulate TAP1, EHD1, GABPA, and IL1B expression in primary murine microglia. **a** Experimental plan for in vivo studies: adult C57BL/6 mice received four daily IP doses of PBS, ShK-223, LPS, or LPS+ShK-223, and brain mononuclear cells were isolated for flow cytometry from one hemisphere and qRT-PCR studies from the other hemisphere (*n* = 3, mice/group). **b** Results from flow cytometric studies measuring intracellular EHD1 protein expression in freshly isolated CNS MPs (gated first on live cells, followed by CD11b^+^ CD45^low^ and CD11b^+^ CD45^high^ populations). At least 10,000 live CNS MPs were counted per sample. *Left:* Example of flow cytometric histograms comparing EHD1 expression in CD11b^+^ CD45^low^ microglia from one mouse from each treatment group. *Right:* Quantitative analysis of EHD1^+^ cells in CD11b^+^CD45^low^ and CD11b^+^CD45^high^ populations. **c** Results from pRT-PCR studies measuring mRNA expression of TAP1, EHD1, GABPA, and IL1B in CNS MPs. For these studies, all CNS MPs isolated from one hemisphere were used for RNA extraction in Trizol, followed by cDNA preparation followed by quantitative PCR. HPRT was used as the housekeeping gene. Data normalized to HPRT were then normalized to PBS-treated control samples (*p < 0.05, **p < 0.01, ***p < 0.005).

Our proteomic findings were derived from BV2 microglia, an immortalized cell line that has been found not to adequately represent primary microglia [34]. Therefore, we performed validation studies of our proteomic findings in freshly isolated CNS MPs from wild-type adult mice what were treated with four daily IP doses of either PBS, ShK-223, LPS, or LPS+ShK-223 (Fig. 5a). One-third of CNS MPs were used for flow cytometry to detect EHD1 along with CD11b and CD45 while the rest were used qRT-PCR experiments (Fig. 5a). In flow cytometric studies of permeabilized cells, we found that EHD-1 was strongly upregulated in the CD11b^+^ CD45^low^ microglial population of CNS MPs and ShK-223 inhibited this upregulation (Fig. 5b). ShK-223 treated mice (without LPS exposure) also demonstrated lower proportions of EHD1-positive cells compared to PBS-treated mice. CD11b^+^ CD45^high^ CNS-infiltrating macrophages did not demonstrate any significant changes in EHD1 with LPS or ShK-223 treatment. We were unable to detect TAP1 by flow cytometric methods. In qRT-PCR studies of purified CNS MPs (Fig. 5c), we observed that LPS upregulated the expression of pro-inflammatory gene IL1B while ShK-223 inhibited LPS-induced IL1B upregulation. TAP1 mRNA expression was upregulated by LPS while ShK-223 inhibited this LPS-induced effect; although, EHD1 mRNA expression was not significantly altered across the treatment groups. GABPA mRNA also demonstrated ShK-223-induced upregulation in CNS MPs; although, LPS did not alter GABPA mRNA expression in CNS MPs. Overall, these validation studies at the protein and mRNA levels using freshly isolated primary microglia validate the top Kv1.3 channel-dependent protein expression changes observed in BV2 cells (TAP1, EHD1, and GABPA).

### Kv1.3 channels regulate MHCI trafficking and MHCI-restricted antigen presentation by LPS-activated microglia

**Fig. 6:**
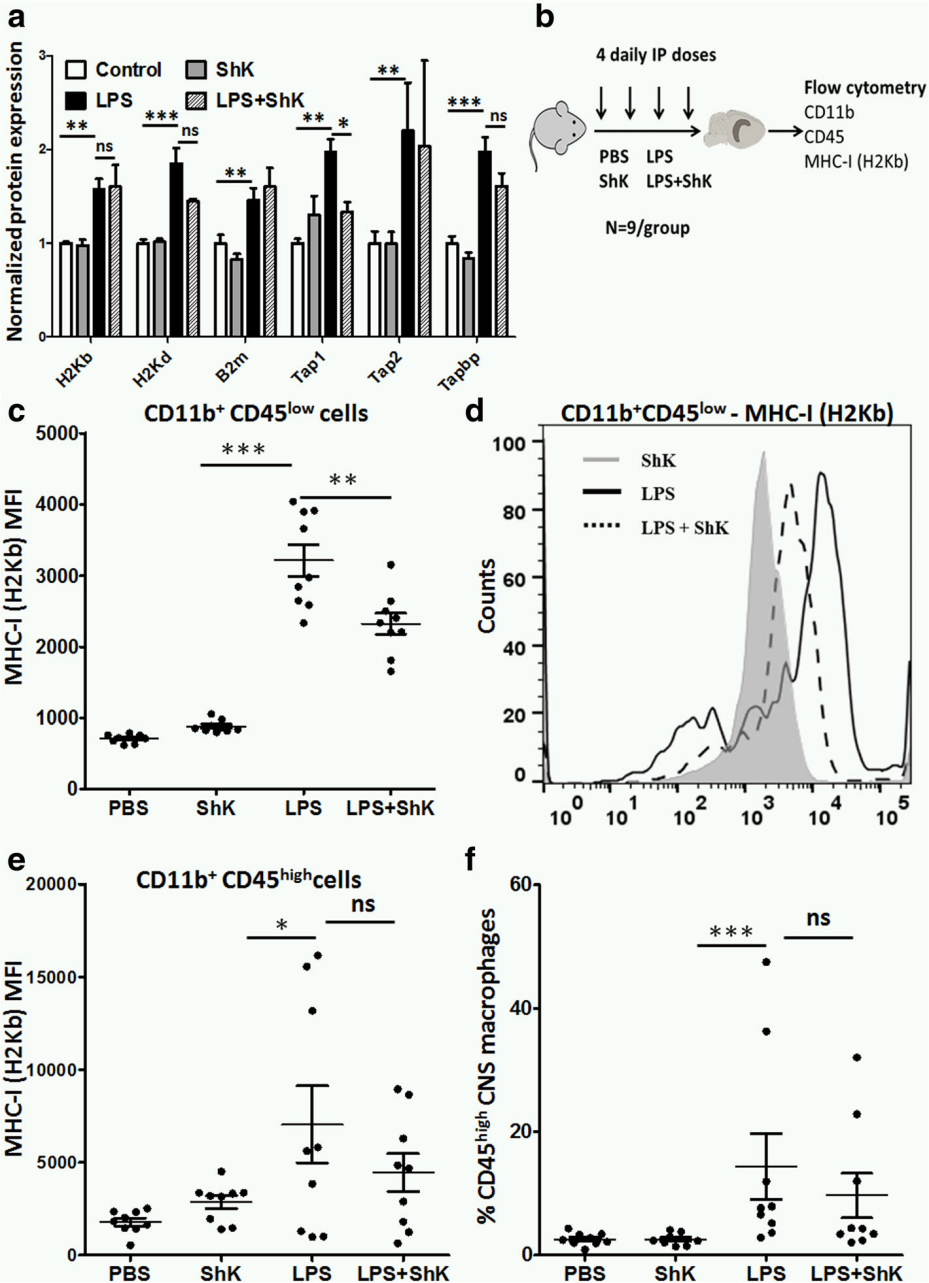
LPS-induced MHCI trafficking in microglia involves Kv1.3 channels. **a** Comparison of MHCI and MHCI-related protein expression in BV2 proteomic data. Normalized LFQ data (compared to control-treatment) is shown (three replicates/group). **b** Experimental plan for in vivo studies: Adult C57BL/6 mice received four daily IP doses of PBS, ShK-223, LPS, or LPS+ShK-223 and brain mononuclear cells were isolated for flow cytometry (pooled *n* = 9/group). **c** Comparison of MHCI (H2Kb) expression (median fluorescence intensity) in CD11b^+^ CD45^low^ microglia. **d** Representative flow cytometry frequency histograms of MHCI expression in CD11b^+^ CD45^low^ microglia. **e** Comparison of MHCI expression in CD11b^+^CD45^high^ brain-infiltrating macrophages. **f** Comparison of the proportions of CD11b^+^CD45^high^ CNS macrophages (among all CD11b^+^ cells) in the four treatment groups (*p < 0.05, **p < 0.01, ***p < 0.005)

Among the Kv1.3 channel-regulated proteins, EHD1 and TAP1 demonstrated the highest LPS upregulation. TAP1, along with TAP2, forms a pore in the endoplasmic reticulum membrane that allows processed antigenic peptides to enter the ER and assemble with MHCI [35]. Prior work has also shown that knockdown of TAP1 also inhibits LPS-mediated MHCI expression by macrophages [36]. Tapasin (TAPBP), also upregulated by LPS but is not affected by ShK-223 in our experiments, facilitates this process [37]. Antigen-MHCI complexes are then transported to the cell surface via a process that involves EHD1 [38]. Therefore, we hypothesized that LPS-induced TAP-1/EHD-1 upregulation facilitates MHCI trafficking and antigen presentation to CD8^+^ T cells in an antigen-specific manner and that Kv1.3 blockade inhibits these processes. We observed that MHCI proteins (H2Kb, H2D, and β2 microglobulin as well as TAP1, TAP2, and TAPBP were upregulated by LPS in our proteomics experiments; although, only TAP1 showed reversal by ShK-223 (Fig. 6a). In flow cytometric studies of LPS-treated BV2 microglia, MHCI surface expression was also increased; although, ShK-223 did not impact MHCI expression. The lack of an effect of ShK-223 on surface MHCI in BV2 cells may have been a result of the persistent low-level of activation of BV2 microglia. A saturating effect of LPS on MHCI protein synthesis may have also overshadowed any effect on MHCI assembly and trafficking. In order to overcome this limitation of our in vitro BV2 model, we tested this hypothesis in vivo. Adult C57BL/6 mice were given four daily intraperitoneal injections of PBS, ShK-223, LPS, or LPS+ShK-223 (Fig. 6b). Flow cytometric studies of brain mononuclear cells showed that MHCI expression in CD11b^+^CD45^low^ microglia was increased following LPS treatment while ShK-223 inhibited this effect (Fig. 6c–d). This inhibitory effect of ShK-223 was not significant in CD45^high^ brain-infiltrating macrophages (Fig. 6e). LPS also significantly increased the proportion of CD45^high^ brain-infiltrating macrophages in the brain and ShK-223 appeared to partly inhibit this LPS effect (Fig. 6f). In summary, these in vivo data confirm that LPS-induced MHCI upregulation in microglia, but not brain-infiltrating macrophages, is a Kv1.3 channel-dependent process.

**Fig. 7:**
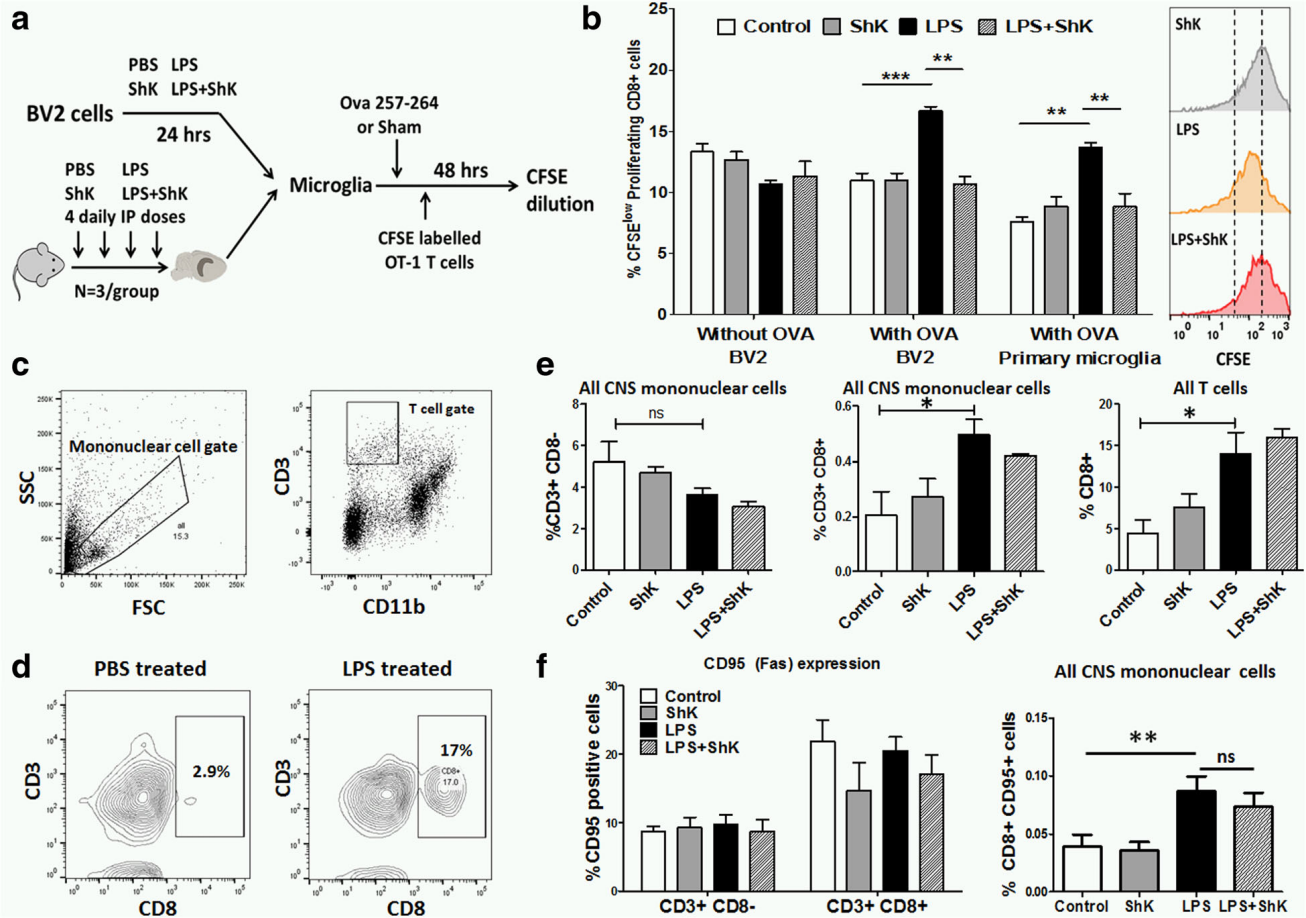
Kv1.3 blockade inhibits LPS-induced MHCI-restricted antigen presentation to CD8 T cells. **a** Experimental plan for MHCI-restricted T cell proliferation studies: BV2 microglia were treated in vitro with PBS, ShK-223 (100 nM), LPS (100 ng/mL), or LPS+ShK-223 for 24 h (four replicates/group) and then loaded with 2 μg/mL Ova (257-264) peptide for 30 min at 37 °C. Washed microglia were co-cultured with CFSE-labeled splenic T cells from Ova (257-264)-specific OT-1 mice for 48 h (25,000 microglia:150,000 T cells/well). For in vivo experiments, adult wild-type mice were treated with four daily IP doses of saline, ShK-223, LPS, or LPS+ShK-223 (*n* = 6/group) and isolated brain mononuclear cells were used for in vitro T cell proliferation studies. After 48 h, CFSE dilution (leftward shift in CFSE staining) was assessed by flow cytometry as a measure of CD8^+^ T cell proliferation. **b** Comparison of proliferating CD8^+^ cells across various treatment groups from in vitro and in vivo studies. *Right*: Representative frequency histograms from in vivo experiments. No direct effects of LPS or Ova peptide on T cells were noted without the presence of microglia. **c** Flow cytometric detection of CD3^+^ CD11b^−^ T cells in brain mononuclear cells after thorough cardiac perfusion. Mice were injected (IP, once daily for 4 days) with PBS, LPS, ShK-223, or LPS+ShK-223. Panel **a** shows the gating strategy. After forward and side-scatter gating for live mononuclear cell population, CD11b^−^ CD3^+^ cells were gated and assessed for T cell markers (CD3, CD8) and activation/cytolytic marker CD95 (Fas). **d** Flow cytometric evidence for increased CD8^+^ T cell trafficking to the brain following LPS treatment. **e** Comparison of proportions of brain CD8^+^ and CD8^−^ T cell populations across the 4 treatment groups. **f** Comparison of CD95 (Fas) expression in brain CD8^+^ and CD8^−^ T cell populations across treatment groups (*p < 0.05, **p < 0.01, ***p < 0.005)

Unstimulated microglia, compared to peripheral macrophages, have lower antigen-presenting capacity which is significantly increased during inflammation [39, 40]. Based on our results, we hypothesized that induction of MHCI may augment microglial antigen presentation capacity to CD8^+^ T cells in a MHCI-restricted manner, resulting in more effective activation and proliferation of cytotoxic CD8^+^ T cells, a response that can be abrogated by Kv1.3 channel blockade. To test this hypothesis, we performed MHCI-restricted T cell proliferation assays using in vitro and in vivo models (Fig. 7a). We observed that LPS-activated and ovalbumin (Ova)-loaded BV2 microglia increased proliferation of Ova-specific CD8^+^ T cells from Ova-specific OT-1 mice, and this increase in proliferation was inhibited by Kv1.3 channel blockade (Fig. 7b). Similarly, microglia isolated from LPS-treated mice also increased Ova-specific T cell proliferation, which was also inhibited by Kv1.3 blockade (Fig. 7b). Supporting the relevance of our observed increased antigen presentation capability of pro-inflammatory microglia, we found that LPS-induced neuroinflammation involves a specific increase in CD8^+^ T cell recruitment to the brain in mice treated (Fig. 7c–e). As compared to CD8^−^ T cells in the brain, CD95 (Fas) expression was also significantly higher in CD8^+^ T cells, but LPS or ShK-223 did not alter CD95 expression in this subpopulation (Fig. 7f). This selectively increased CD8^+^ T cell trafficking to the brain in response to LPS treatment suggests that the Kv1.3-dependent upregulation of MHCI by microglia can impact neuroinflammatory responses of CD8^+^ cytotoxic T cells.

### Kv1.3 channel regulates signaling downstream of LPS

**Fig. 8:**
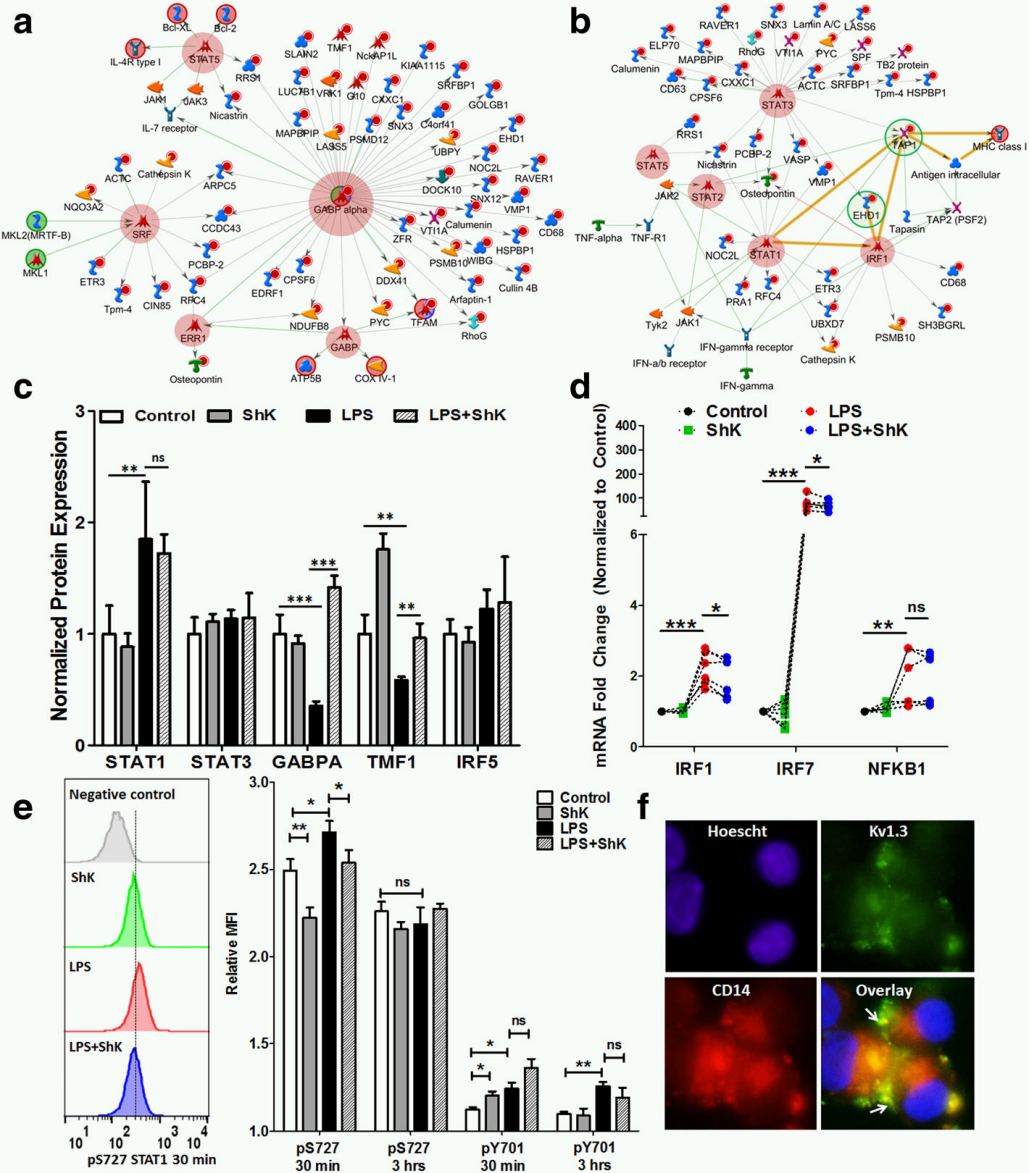
Identification of Kv1.3-regulated signaling mechanisms in LPS-induced microglial activation. **a**, **b** Canonical pathway analysis of 120 Kv1.3-dependent proteins identified in our proteomic dataset revealed highly represented signaling pathways, two of which are shown here (see Additional file 2: Table S5 for others). Members of this list of 120 proteins are marked with (*red circle*). Transcription factors are also highlighted (*transparent red circle*). *Arrows* indicate directionality of the interaction (upstream vs. downstream). **a** GABPA, a Kv1.3-dependent transcription factor that was downregulated by LPS, was placed upstream of several Kv1.3-regulated proteins. **b** MHCI proteins of relevance to our results (TAP1, Tapasin, and EHD1 proteins) were represented in a signaling network that suggested that STAT1 and IRF1 may serve as upstream regulators of these proteins. **c** Comparison of normalized protein expression of transcription factors identified in our proteomic dataset across treatment groups. **d** Quantitative RT-PCR data comparing IRF1, IRF7, and NFKB1 mRNA expression across treatment groups (paired *t* tests were used for these comparisons; six replicates/group). **e** Phospho-flow cytometric studies of serine (S727) and tyrosine (Y701) STAT1 phosphorylation in BV2 microglia (*n* = 5/treatment group) at 30-min and 3-h time points. **f** Immunofluorescence microscopy showing partial co-localization between Kv1.3 (*green*, detected by ShK-F6CA labeling) and CD14 (*red*) in LPS-activated BV2 microglia (*p < 0.05, **p < 0.01, ***p < 0.005)

While our results identified several novel proteins whose regulation is downstream of Kv1.3 channel function, the specific signaling mechanisms regulated by Kv1.3 channels in microglia are not defined. Using our proteomic dataset, we first performed canonical pathway analyses on a list of 120 BV2 microglial proteins whose expression was significantly altered by Kv1.3 channel blockade with or without LPS activation and identified the putative and most highly represented signaling pathways (Additional file 2: Table S4). The top two identified pathways contained 61 and 52 Kv1.3-regulated proteins respectively, and several of which were downstream of GABPA, CREB1, and SRF transcription factors (Fig. 8a, Additional file 2: Table S5). The third most significant pathway contained 36 proteins (including TAP1, EHD1, and VASP proteins) which were downstream of STAT1, IRF1, and STAT3 transcription factors (Fig. 8b). Within our proteomic dataset, GABPA itself was downregulated by LPS and reversed by Kv1.3 blockade while STAT1 and STAT3 were not significantly altered by LPS or Kv1.3 blockade (Fig. 8c). The only IRF family protein identified in our proteomic dataset was IRF5, which did not show altered regulation across experimental conditions. Since we validated Kv1.3’s role in MHCI expression, we focused on signaling mechanisms that may regulate TAP1 and EHD1. Transcriptional regulation of TAP1 involves STAT1-signaling, resulting in increased transcription of IRF1, which in turn binds to an IRF1 binding site in the TAP1 promoter region to induce TAP1 transcription [41]. IRG1, another strongly upregulated protein in LPS-activated microglia that was partly inhibited by ShK-223 in our experiments, is also downstream of IRF1 signaling [42]. We therefore hypothesized that IRF1 upregulation by LPS can be inhibited by ShK-223. In qRT-PCR experiments, we observed that the nearly twofold upregulation of IRF1 and IRF7 by LPS1 was partially inhibited by ShK-223, while NFKB1 upregulation was not affected by ShK-223 (Fig. 8d). Since STAT1 protein expression was unaltered in our proteomic dataset but STAT1 is a known common regulator of TAP1, EHD1, and other proteins (Fig. 8b), we also hypothesized that STAT1 phosphorylation may be regulated by Kv1.3 channel function. Serine phosphorylation of STAT1 (S727) is directly induced by LPS-TLR signaling and by IFNγ [43], while tyrosine phosphorylation of STAT1 (Y701) is an IFNγ-dependent process that involves autocrine IFNγ signaling. In phospho-flow cytometric studies of BV2 microglia probing for S727 and Y701 STAT1 phosphorylation, we found that LPS increased S727 STAT1 phosphorylation at 30 min, a response that was partially inhibited by ShK-223 while no LPS or ShK-223 effects were seen at 3 h (Fig. 8e). Interestingly, ShK-223 also inhibited baseline levels of S727 STAT1 phosphorylation, reflecting baseline level of activation in BV2 microglia. Y701 STAT1 phosphorylation was slightly induced by LPS at 30 min and 3 h; although, ShK-223 did not alter the LPS effect. Since LPS binds to the cell surface receptor complex that is comprised of CD14 and toll-like receptors (TLRs) [44], we hypothesized that Kv1.3 channels must also lie in proximity to the CD14-TLR receptor complex in activated microglia in order to regulate early signaling events triggered by LPS such as STAT1 phosphorylation. Consistent with this hypothesis, we observed that Kv1.3 channels (labeled by ShK-F6CA) partly co-localized with CD14 in LPS-activated BV2 cells (Fig. 8f).

## Discussion

**Fig. 9:**
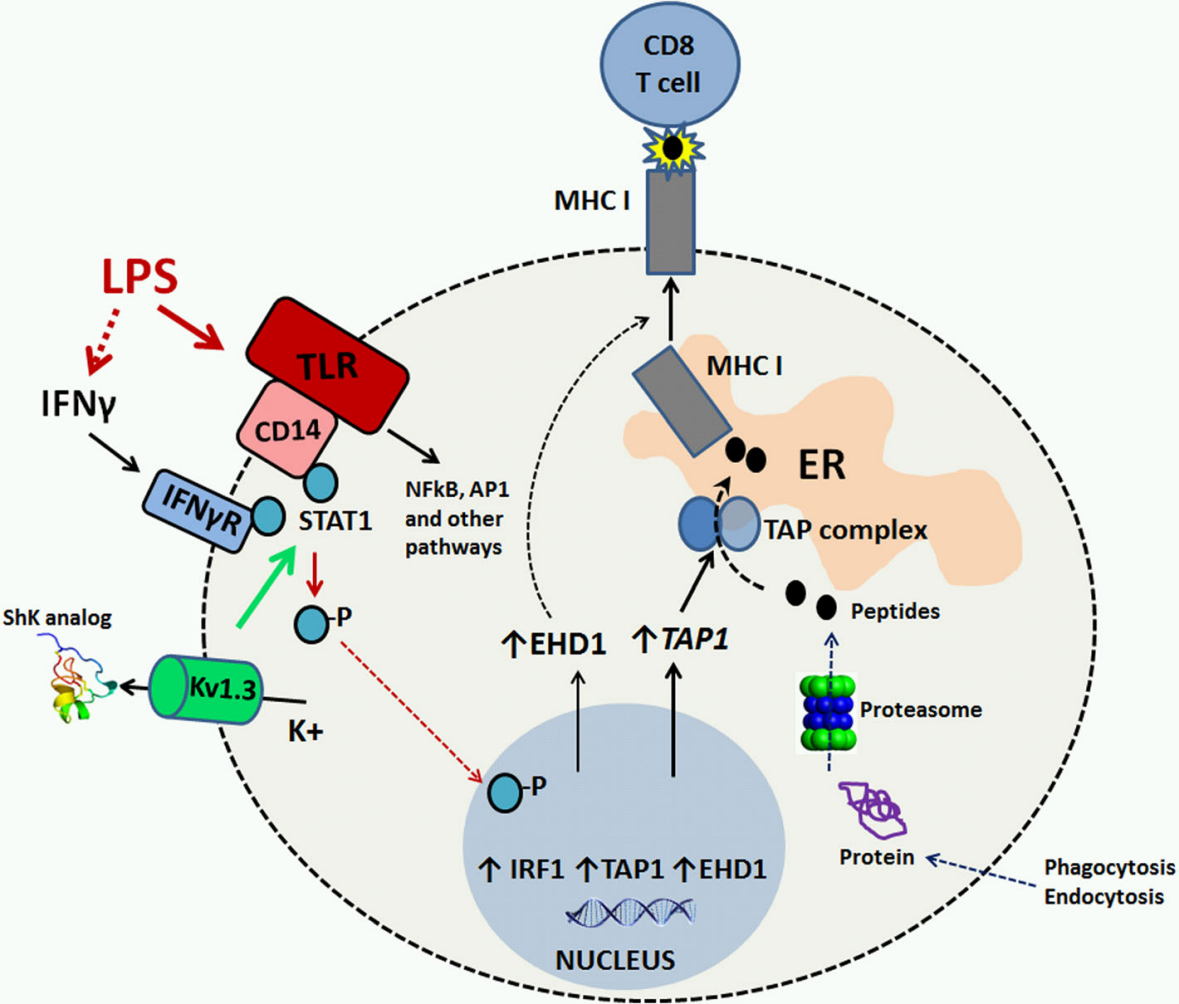
Cartoon showing the proposed mechanism by which Kv1.3 channel function regulates LPS-induced EHD1 and TAP1 upregulation and MHC I trafficking an antigen presentation by microglia

We provide confirmatory in vivo and in vitro evidence for upregulation and functional importance of Kv1.3 channels during pro-inflammatory activation of microglia. We used LPS as the prototypic immune stimulus that induces systemic and neuroinflammatory responses that recapitulate microglial activation seen in chronic neuroinflammatory and neurodegenerative states [26, 27]. Our proteomics-based approach has unraveled previously novel Kv1.3-dependent molecular mechanisms involved in pro-inflammatory microglial activation, including microglial chemotaxis and focal adhesion formation. We have also found that Kv1.3 channels regulate antigen-presenting capacity of activated microglia. Specifically, Kv1.3 channel function underlies upregulation of TAP1 and EHD1, both of which regulate MHCI assembly and trafficking to the cell surface. Inhibition of Kv1.3 reduces LPS-induced TAP1 and EHD1 protein expression and inhibits MHCI trafficking to the cell surface, resulting in decreased antigen presentation to CD8^+^ T cells and decreased T cell proliferation in a MHCI-restricted manner. The relevance of this novel Kv1.3 channel-dependent mechanism is reflected by the increased trafficking of CD8^+^ but not CD8^−^ T cells to the acutely inflamed brain, providing the opportunity for microglia with upregulated MHCI to interact with CD8^+^ T cells and thereby regulate their effector or regulatory functions. Our results also suggest that Kv1.3 channels regulate LPS-induced serine phosphorylation (S727) but not tyrosine phosphorylation (Y701) of STAT1, as well as IRF1 expression, as early signaling events following LPS activation. The functional coupling between Kv1.3 channels and LPS-TLR signaling in activated microglia is also suggested by the co-localization of Kv1.3 channels with the CD14-TLR complex. Based on these findings, we present a cohesive model (Fig. 9) suggesting that Kv1.3 channels assemble with the CD14/TLR complex on microglia during LPS activation and regulate S727 phosphorylation of STAT1, which in turn regulates transcription of TAP-1 and EHD-1 and regulates MHCI assembly and trafficking of the MHCI-antigen complex to the cell surface of microglia. This upregulation of MHCI trafficking and/or assembly with antigen in pro-inflammatory microglia improves their ability to present antigens to CD8^+^ T cells within the inflamed brain.

The regulation of microglial MHCI expression and antigen presentation highlight novel roles for microglial Kv1.3 channels in adaptive immune responses mediated by CD8^+^ T cells in the brain. T cell-mediated responses involve both CD4^+^ and CD8^+^ T cells, and the pathogenic roles of CD8^+^ T cells in neuroinflammatory diseases such as multiple sclerosis and neurodegenerative diseases are being increasingly recognized [45, 46]. We found evidence for increased CD8^+^ T cell traffic to the brain in response to systemic inflammation, and a large proportion of these cells express CD95 (Fas) suggesting their cytolytic effector properties. The proportion of CD8^−^ T cells in the brain, on the other hand, did not change after LPS treatment. Additionally, microglia-induced CD8^+^ T cell proliferation required Ova peptide (Ova 257-264) which is specific for MHCI but not MHCII. Consistent with our observations, others have also found that CD8^+^ T cell traffic to the brain and their proliferation within the brain are antigen-specific and MHCI-dependent [47]. However, we did not observe an effect of ShK-223 on LPS-induced brain infiltration by CD8^+^ T cells, suggesting that CD8^+^ T cell traffic to the brain does not require Kv1.3 channels. This may be partly explained by the low levels of Kv1.3 channels expressed by most T cells, with the exception of activated CD4^+^ and CD8^+^ effector memory T cells [48, 49]. It is also possible that CD8^+^ T cell traffic to the brain requires antigen-presenting cells other than microglia, such as endothelial cells. Endothelial cells, like microglia, strongly upregulate MHCI in response to systemic inflammation [50] and, unlike microglia, express KCa3.1 potassium channels that are resistant to Kv1.3 blocking ShK peptides [4, 51]. These findings support the possibility that MHCI upregulation by activated microglia, a Kv1.3 channel-dependent process, may modulate proliferative and cytotoxic responses of CD8^+^ T cells after they enter the brain. Since CD8^+^ T cells can adopt cytotoxic and regulatory functions, further characterization of CD8^+^ T cell subsets in the brain needs to be performed [52]. Our observations provide a rationale for testing microglial Kv1.3 blocking strategies as a novel therapeutic approach to modulate adaptive immune responses mediated by CD8^+^ T cells in neurological diseases.

Existing literature support the candidacy of microglial Kv1.3 channels as therapeutic targets in neurologic diseases [6]. Kv1.3 channels are highly expressed by microglia in the brains of AD patients as compared to non-AD controls [7]. Microglial Kv1.3 channels are also upregulated following status epilepticus, in radiation neurotoxicity and HIV-TAT neurotoxicity models, and inhibition of Kv1.3 channels in these models has demonstrated anti-inflammatory and neuroprotective effects [53–55]. Our observation of CD14 and Kv1.3 channel co-localization may also be highly relevant to neuro-infectious, neuroinflammatory, and neurodegenerative disorders, where TLR activation has pathologic relevance [55, 56]. Our results provide in vivo evidence for Kv1.3 upregulation in a model of acute neuroinflammation in addition to providing mechanistic insights into the roles of Kv1.3 channels in pro-inflammatory microglial activation. ShK analogs with high selectivity for Kv1.3 channels are the most potent blockers of this channel, with excellent safety profiles in animals and recently completed phase II human studies [57]. A potential limitation of peptide-based therapeutics for neurologic diseases, where microglia mediate neuroinflammation, is their anticipated low CNS bioavailability. Increased BBB permeability has been reported in rodent models of neurodegeneration as well as in neuroinflammatory states [58] and may allow for CNS entry of small peptidergic drugs. Our data provide indirect yet compelling evidence for the ability of systemically administered Kv1.3 blocking ShK peptides to inhibit MHCI upregulation by microglia without altering the activation and recruitment of peripherally derived macrophages or CD8^+^ T cells to the brain. The ability of peripherally administered ShK-223 to modulate microglial activation may result from two possible mechanisms: (1) ShK-223 may either affect intermediary cell types in the peripheral immune system which in turn produce factors that enter the brain and activate microglia or (2) ShK-223 is able to enter the brain following LPS treatment and directly modulate microglia. Further studies are needed to clarify these possibilities. We used ShK-223 for our experiments based on its higher selectivity and improved stability profiles as compared to ShK-186 (dalazatide) that is being currently tested in humans [57]. Since ShK-186 is a very well-characterized Kv1.3 blocker, we confirmed that our in vitro observations with ShK-223 were mirrored by ShK-186. Furthermore, binding of ShK-F6CA to microglia was also diminished by pre-incubation with ShK-biotin. These data confirm that our observed results are indeed a result of Kv1.3 channel blockade.

LPS, a classical pro-inflammatory activator of microglia and macrophages, directly binds to the TLR-CD14 complex and induces several downstream signaling cascades. LPS activation also involves indirect autocrine and paracrine effects of IFNγ and TNF [26, 43, 59]. Our observations support the early regulation of STAT1 S727 phosphorylation by Kv1.3 channels, a signaling event that can result from direct LPS-TLR signaling mediated through p38 MAP kinase activity as well as by IFNγ receptor activation by paracrine or autocrine IFNγ [59]. Since peripherally administered LPS has not been found to substantially cross the blood–brain barrier even with repeated dosing, intermediaries such as brain-infiltrating immune cells or cytokines (TNF) that cross the blood–brain barrier may be involved in microglial activation through non-TLR-based mechanisms [26, 60]. While the small amounts of LPS that cross the blood–brain barrier after systemic administration may be adequate for TLR activation, this seems unlikely to account for the robust microglial activation observed in our model. Since we found higher proportions of brain-infiltrating macrophages as well as CD8^+^ T cells in LPS-treated mice, it is possible that these cells contribute to microglial activation and MHCI upregulation. Since Kv1.3 blockade did not alter LPS-induced trafficking of CD8^+^ T cells or CD45^high^ macrophages to the brain and did not alter MHCI expression by peripherally derived macrophages in the brain, the observed effects of ShK-223 on microglial MHCI upregulation must be mediated by intermediary factors instead. The lack of an effect of Kv1.3 channel blockade on brain-infiltrating macrophages despite their higher expression of Kv1.3 channels also suggests that alternative K channels such as Kir2.1, KCa3.1, and K-ATP channels and possibly other cationic (TRP) channels may play more important roles in peripherally derived macrophages as compared to microglia [61–63]. Since our studies focused on Kv1.3 channels, we are unable to exclude any contribution of alternative cationic channels to MHCI upregulation in microglia.

In addition to the Kv1.3-regulated STAT1-IRF1-TAP1/EHD1-MHCI antigen presentation pathway highlighted in this manuscript, we have identified several novel putative molecular pathways that may be regulated by Kv1.3 channels in microglia, including roles for Kv1.3 channels as regulators of RNA processing, mitochondrial biogenesis, and nuclear function. Of particular interest is the transcription factor GABPA, a subunit of the DNA (GA) binding subunit of protein transcription factor GABP that is predicted to regulate transcription of over 50 Kv1.3-regulated proteins. We found that Kv1.3 blockade upregulates GABPA expression in both BV2 microglia and primary microglia. GABP (NRF2) is a key transcriptional regulator of mitochondrial proteins involved in oxidative phosphorylation and mitochondrial DNA replication, transcription, and translation [64]. In addition to being expressed on the cell surface, functional Kv1.3 channels are also expressed in the mitochondria membrane where they regulate mitochondrial membrane potential and oxidative burst and blockade of mitochondrial Kv1.3 channels inhibits neurotoxicity mediated by activated microglia [9, 65]. While our studies primarily tested the effect of blockade of cell surface Kv1.3 channels by a membrane non-permeant peptide blocker of Kv1.3 channels, it may be possible that extracellular ShK-223 is endocytosed by microglia and released intracellularly, allowing the peptide to interact with intracellular Kv1.3 channels. Independent of this possibility, our results suggest a novel pathway for regulation of mitochondrial biogenesis by Kv1.3 channels mediated through GABPA.

A few caveats about our study merit consideration. Since only 3141 proteins were identified in our proteomic studies, some Kv1.3 channel-regulated proteins may not have been identified. Secreted proteins including cytokines were also likely not identified using our approach. Another limitation is the fact that our studies focused primarily on LPS as the activating stimulus. LPS has pleitropic effects on TLRs 1-4 that result in distinct and overlapping downstream signaling cascades; these were not examined in our studies. Although we are unable to confirm the functional coupling between a specific TLR and Kv1.3 channels, we provide supportive evidence for Kv1.3 co-localization with CD14, a key member of the LPS-TLR receptor complex. While LPS-induced acute neuroinflammation is a well-established method to induce microglial activation, we cannot be certain that our observed in vivo or in vitro effects were results of direct LPS-TLR interaction as opposed to indirect autocrine effects of pro-inflammatory cytokines such as IFNγ or TNF. Since we observed an inhibition of STAT1 S727 phosphorylation by ShK-223 within 30 min of LPS exposure, we suspect that Kv1.3 channels regulate early signaling events following LPS-TLR binding but other effects of late responses cannot be excluded. Future studies will focus on investigating the extent to which other activating stimuli for microglia rely on the function of Kv1.3 and will reveal the universality of the mechanism described herein.

## Conclusions

Using a systems pharmacology approach, we have unraveled novel molecular and functional roles for Kv1.3 channels in pro-inflammatory microglial activation, including a Kv1.3 channel-regulated pathway that facilitates MHCI expression and MHCI-dependent antigen presentation by microglia to CD8^+^ T cells. We also provide evidence for neuro-immunomodulation by systemically administered ShK peptides. Our results further strengthen the therapeutic candidacy of microglial Kv1.3 channels in neurologic diseases.

## Additional files

Additional file 1: Figures S1–S6. Flow cytometric confirmation of Kv1.3-channel specificity of the ShK-F6CA binding assay is shown in **Figure S1.** Comparison of ShK-F6CA labeling of Kv1.3 channels in splenic and brain-infiltrating macrophages is shown in **Figure S2.** Morphological changes induced by LPS, ShK-223, and LPS+ShK-223 treatment conditions are shown in **Figure S3.** Distribution of missing data in the proteomic data set is shown in **Figure S4.** Quantitative RT-PCR data showing validation of pro-inflammatory activation of BV2 microglia by LPS are shown in **Figure S5.** EHD1 upregulation by LPS and inhibition of EHD1 upregulation by ShK-223 is shown in Figure S6. (DOCX 1262 kb)

Additional file 2: Tables S1–S5. Raw LFQ expression data from proteomic experiments are provided in Supplemental **Tables S1** and **S2.** **Table S3** lists LPS-regulated proteins in our analysis, cross-referenced with previously published microglia proteomics and transcriptomics data. **Table S4** contains lists of all Kv1.3-dependent proteins identified in this study. **Table S5** contains results from the canonical pathway analysis of Kv1.3-dependent proteins. Raw proteomic datasets have been deposited at http://www.proteomexchange.org/. (XLSX 1848 kb)

## Abbreviations

DCFDA: Dichlorofluorescein diacetate
EHD1: EH domain containing protein I
GO: Gene ontology
LPS: Lipopolysaccharide
MHCI: Major histocompatibility complex I
MS: Mass spectroscopy
PBS: Phosphate-buffered saline
ROS: Reactive oxygen species
STAT1: Signal transducer and activator of transcription 1
TAP1: Transporter associated with antigen processing 1
TLR: Toll-like receptor
