# Supplementary material for: A systems pharmacology-based approach to identify novel Kv1.3 channel-dependent mechanisms in microglial activation
Source: J Neuroinflammation. 2017 Jun 26;14:128. doi: 10.1186/s12974-017-0906-6 (PMC5485721; doi:10.1186/s12974-017-0906-6)
Supplement: Supplementary file 1 — Flow cytometric confirmation of Kv1.3-channel specificity of the ShK-F6CA binding assay is shown in Figure S1. Comparison of ShK-F6CA labeling of Kv1.3 channels in splenic and brain-infiltrating macrophages is shown in Figure S2. Morphological changes induced by LPS, ShK-223, and LPS+ShK-223 treatment conditions are shown in Figure S3. Distribution of missing data in the proteomic data set is shown in Figure S4. Quantitative RT-PCR data showing validation of pro-inflammatory activation of BV2 microglia by LPS are shown in Figure S5. EHD1 upregulation by LPS and inhibition of EHD1 upregulation by ShK-223 is shown in Figure S6. (DOCX 1262 kb) [file 12974_2017_906_MOESM1_ESM.docx]

**A systems pharmacology based approach to identify novel Kv1.3 channel dependent mechanisms in microglial activation**

Srikant Rangaraju,^1^ Syed Ali Raza,^1^ Andrea Pennati,^2^ Qiudong Deng,^3^ Eric B. Dammer,^1^ Duc Duong,^3^ Michael W. Pennington,^4^ Malu G. Tansey, ^5^ James J. Lah,^1^ Ranjita Betarbet,^1^ Nicholas T. Seyfried,^3^ Allan I. Levey^1^

^1^Department of Neurology, Emory University; ^2^Department of Medicine (Hematology-Oncology), University of Wisconsin, Madison; ^3^Department of Biochemistry, Emory University; ^4^Peptides International, Louisville, Kentucky; ^5^Department of Physiology, Emory University

Corresponding author: Srikant Rangaraju

Address: 615 Michael Street, Suite 525, Center for Neurodegenerative Diseases, Emory University, Atlanta, GA 30322

Email address: [srangar@emory.edu](mailto:srangar@emory.edu) Telephone number: 404-727-0633

**Supplemental Data**

Supplemental Figures: 6 Supplemental Tables: 5


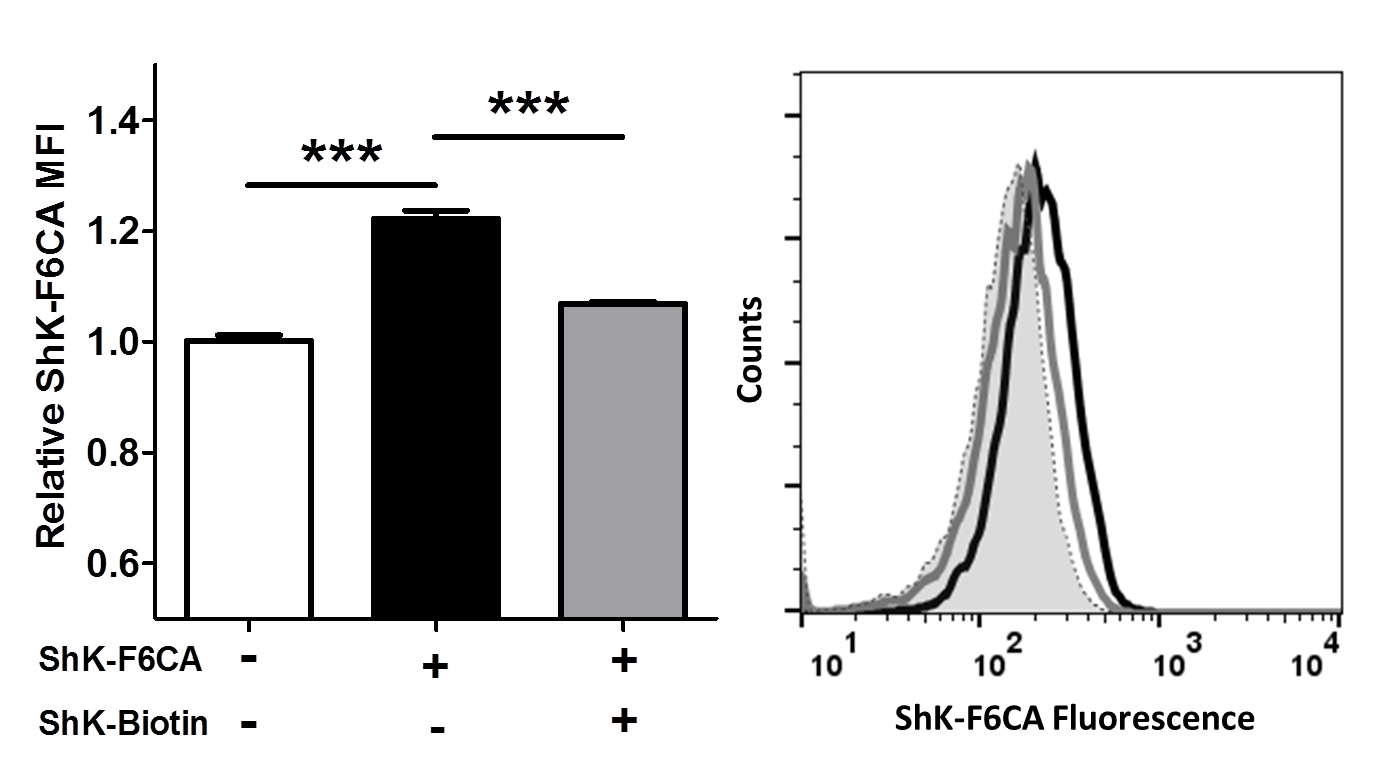


**Supplemental Figure S1. Competitive inhibition of ShK-F6CA labelling of Kv1.3 channels by non-fluoresceinated ShK-biotin.** BV2 microglia activated by LPS were either incubated with 10 nM ShK-F6CA (IC_50_ ≈ 25 pM) or first pre-incubated with 100 nM ShK-Biotin (IC_50_ ≈ 49 pM) followed by 10 nM ShK-F6CA. ShK-F6CA labelling of Kv1.3 channels was measured by flow cytometry. Relative Median Fluorescence Intensity (MFI) was compared across groups (Left: *** p<0.005, 3 replicates/group). Representative frequency histograms from flow cytometric experiments are shown (Right: Black line – Without ShK-biotin pre-incubation, Grey line - With ShK-biotin pre-incubation, Dotted line: Unstained control).

**
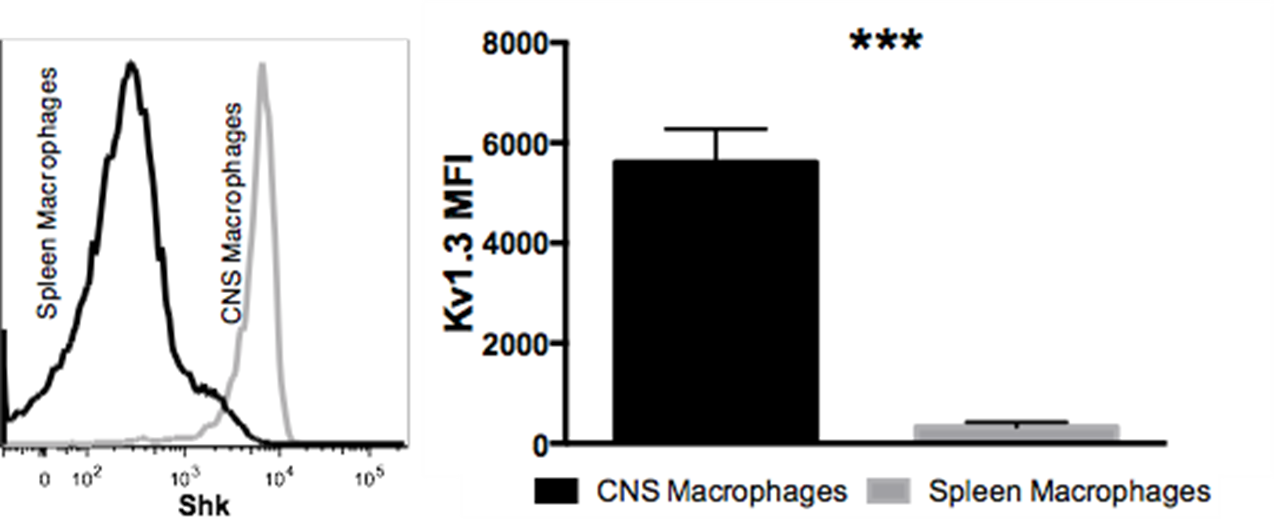
**

**Supplemental Figure S2. Low Kv1.3 channel expression by splenic macrophages as compared to CNS-infiltrating macrophages.** In WT C57B6/L mice (n=3/group), cell surface Kv1.3 channel expression detected by ShK-F6CA labelling, was significantly lower in splenic CD11b^+^ CD45^high^ macrophages as compared to CD11b^+^ CD45^high^ macrophages isolated from the brain.


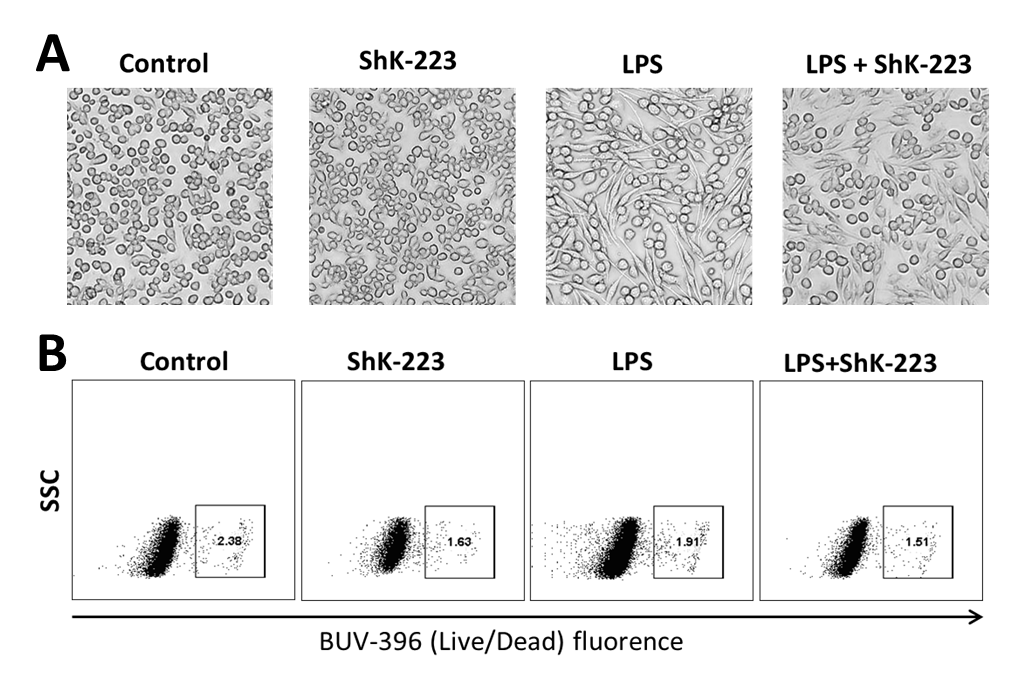


**Supplemental Figure S3. Morphological changes in BV2 microglia induced by LPS at 24 h. (A)** BV2 microglia responded to LPS treatment (100 ng/mL x 24 hours) by becoming larger, more amoeboid and bipolar shaped. This effect appeared to be partly inhibited by ShK-223 treatment (100 nM). ShK-223 alone appeared to have no morphological effects on BV2 microglia. **(B)** Cell viability data from BV2 microglia treated with control, ShK-223 (100nM), LPS (100 ng/mL) or LPS+ShK-223 for 24 hours. In this assay, dead cells are labelled positive for the Live/Dead blue stain. Minimal cell death was observed in the BV2 cell population across all conditions and no within-group differences were observed.


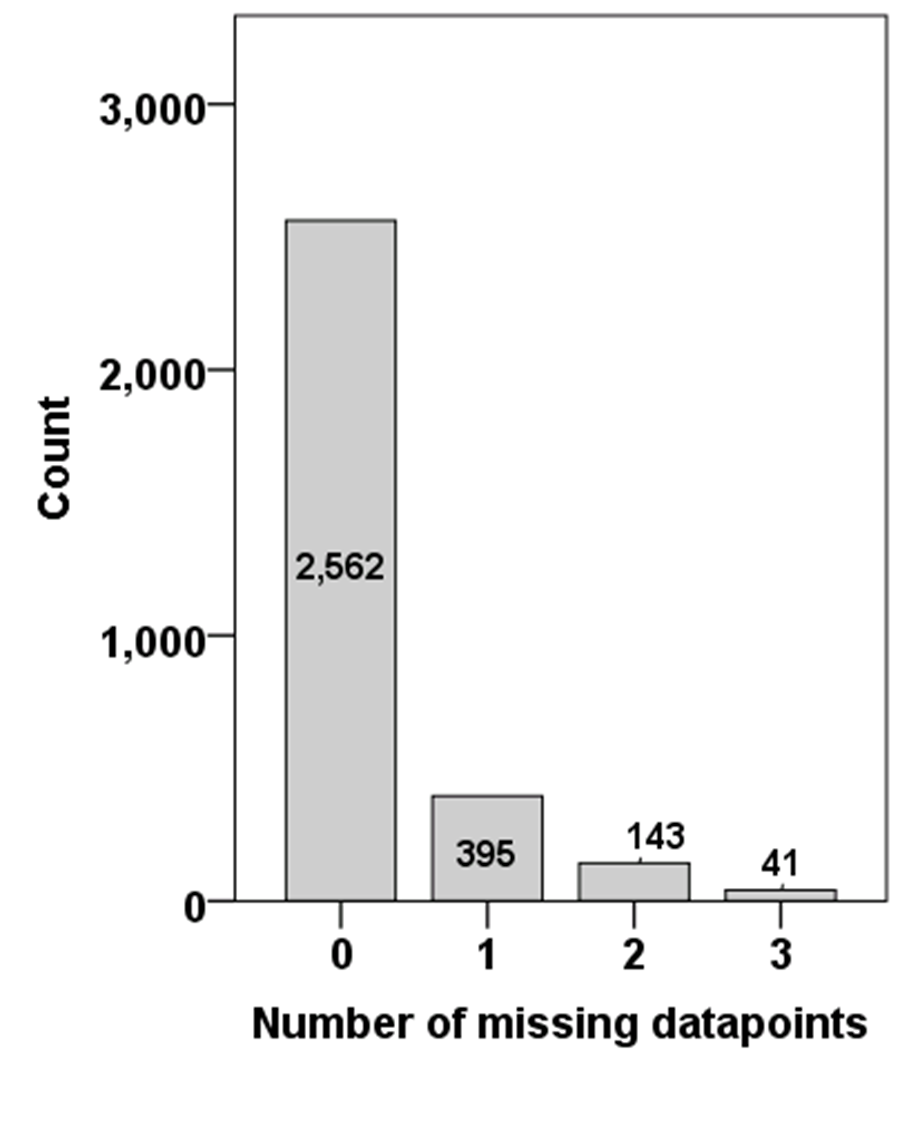


**Supplemental Figure S4. Profile of missing data in the BV2 microglial proteomic dataset.** Of 3141 proteins identified across 4 treatment groups (total N=12) with <25% overall missing data and ≤1 missing data point per treatment group, 2562 (81.6%) proteins had no missing values while only 184 (5.9%) had >1 missing value.

**Supplemental Figure S5. Validation of pro-inflammatory activation of BV2 microglia by LPS.** Results from qPCR studies confirm upregulation of pro-inflammatory cytokine IL1B and immune response genes IRF1 and IRF7 in addition to down-regulation of anti-inflammatory gene ARG1. N=3 biological replicates per condition. BV2 cells were treated with LPS (100 ng/mL) for 24 hours for these experiments. *** p<0.001.


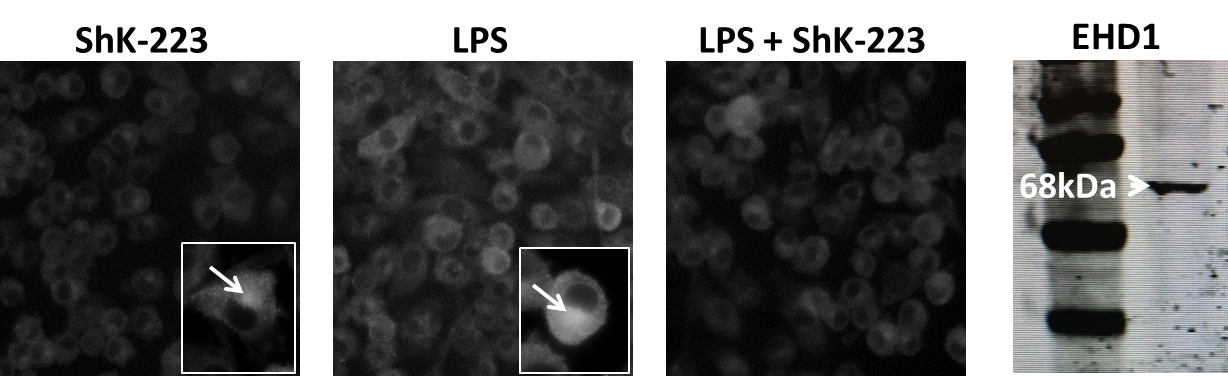


**Supplemental Figure S6. Confirmation of EHD1 upregulation by LPS and reversal by ShK-223.** Immunofluorescence microscopy experiments demonstrated that LPS treatment (100 ng/mL x 24 hours) intracellular upregulated EHD1 expression as compared to untreated control and ShK-223 treated cells. ShK-223 treatment reversed the effect of LPS on EHD1 upregulation. (N=3 biological replicates with 6-8 images per condition). The polyclonal anti-EHD1 rabbit antibody used specifically detected a 68 kDa band in Western Blot experiments, consistent with the predicted molecular weight of EHD1.
